# Modification Strategies for Development of 2D Material‐Based Electrocatalysts for Alcohol Oxidation Reaction

**DOI:** 10.1002/advs.202306132

**Published:** 2023-12-03

**Authors:** Haichang Fu, Zhangxin Chen, Xiaohe Chen, Fan Jing, Hua Yu, Dan Chen, Binbin Yu, Yun Hang Hu, Yanxian Jin

**Affiliations:** ^1^ School of Pharmaceutical and Chemical Engineering Taizhou University Jiaojiang Zhejiang 318000 China; ^2^ Department of Materials Science and Engineering Michigan Technological University Houghton MI 49931 USA

**Keywords:** 2D materials, alcohol oxidation reaction, catalyst supports, electrocatalysts

## Abstract

2D materials, such as graphene, MXenes (metal carbides and nitrides), graphdiyne (GDY), layered double hydroxides, and black phosphorus, are widely used as electrocatalyst supports for alcohol oxidation reactions (AORs) owing to their large surface area and unique 2D charge transport channels. Furthermore, the development of highly efficient electrocatalysts for AORs via tuning the structure of 2D support materials has recently become a hot area. This article provides a critical review on modification strategies to develop 2D material‐based electrocatalysts for AOR. First, the principles and influencing factors of electrocatalytic oxidation of alcohols (such as methanol and ethanol) are introduced. Second, surface molecular functionalization, heteroatom doping, and composite hybridization are deeply discussed as the modification strategies to improve 2D material catalyst supports for AORs. Finally, the challenges and perspectives of 2D material‐based electrocatalysts for AORs are outlined. This review will promote further efforts in the development of electrocatalysts for AORs.

## Graphical abstract

Recent progresses in 2D material‐based electrocatalysts for alcohol oxidation reaction are reviewed with emphasis on modification strategies to improve catalytic performance via tuning 2D material supports, including surface molecular functionalization, heteroatom doping, and composite hybridization.

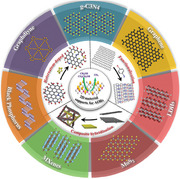

## 1 Introduction

In the past two decades, many attempts have been made in the development of clean energy technologies. Fuel cell energy systems, which convert chemical energy into electrical energy via electrochemical reactions at the electrode surface, are considered to be one of the most promising technologies for solving global energy problems.^[^ ^1^, ^2^, ^3^, ^4^ ^]^ Among many types of fuel cells, direct alcohol fuel cells (DAFCs) have received extensive attention due to the advantages of high energy density (e.g., methanol: 17.28 MJ L^−1^, ethanol: 23.4 MJ L^−1^, and glycerol: 23.04 MJ L^−1^), environmental friendliness, low cost, and portability of alcohols.^[^ ^5^ ^]^ Efficient DAFCs with low production cost and high power density is greatly dependent on anode catalysts with high activity and low cost. To date, platinum (Pt) and palladium (Pd) catalysts have been identified to be the efficient metal catalysts used for DMFCs due to their optimal sorption properties in volcano‐shaped activity trends.^[^ ^6^, ^7^ ^]^ Unfortunately, Pt and Pd nanoparticles (NPs) are susceptible to the toxic effects of CO intermediates during the alcohol oxidation reaction (AOR), leading to a sharp decrease in electrocatalytic activity.^[^ ^8^ ^]^ Furthermore, Pt and Pd resources are scarce and expensive. Therefore, the main goal of research in current AORs has been the design and preparation of high‐performance anode catalysts with low precious metal usage.

Over the past few years, a wide range of strategies has been employed to improve the catalytic performance of precious metal catalysts toward AOR and reduce the catalyst cost, such as alloying with other metals (e.g., ruthenium (Ru), stannum (Sn), nickel (Ni)), formation of material‐supported catalysts with (non)carbon‐based materials.^[^ ^9^, ^10^, ^11^ ^]^ Among them, novel alloy catalysts are prepared by alloying precious metals, which can significantly improve their stability and catalytic activity due to electronic effect and bifunctional mechanism.^[^ ^12^, ^13^, ^14^ ^]^ The electrocatalytic performance of Pt‐ or Pd‐based catalysts was demonstrated to be exhaustively modified and optimized through metal alloying. Another common strategy is to develop supported metal catalysts. On the one hand, the dispersion of active metals on supports can effectively reduce the required amount of precious metals. On the other hand, besides acting as a carrier, a support would have a strong interaction with the active metal component. The catalytic activity primarily depends on the binding energy of the metal active sites to reaction intermediates, which should be neither too strong nor too weak for a superior electrocatalyst, as suggested by the Sabatier principle.^[^ ^15^ ^]^ Furthermore, this binding energy is mainly associated with the d‐band (d orbital on metal atoms) for precious metals‐based electrocatalysts.^[^ ^16^ ^]^ In supported metal catalysts, charge transfer occurs between supports and metals due to their strong electronic interactions, which changes the occupied state of the d‐orbital of the metal and impacts the adsorption, activation, and desorption of reaction intermediates in the oxidation reaction with improved catalytic activity. Thus, the materials used as supports for NPs play an important role in the activity of catalysts, which providing new design opportunities to develop efficient anode catalysts for AORs.

2D nanomaterial has attracted much attention with the successful isolation of single atomic layer graphite (graphene). A distinctive feature of these materials is that one dimension is an outside the nanoscale and the other two dimensions are only one or several atoms thick. Atoms in the planes are connected by strong covalent bonds, while weak van der Waals interactions exist between layers. As a result, these 2D structured materials have many unique properties compared to other structures, such as large surface area, ease of charge migration and mass transfer, and unique 2D charge transport channels.^[^ ^17^ ^]^ Considering these structural and chemical properties of 2D materials, many researchers have gradually paid much attention in develop supported metal catalysts with excellent electrocatalytic performance based on 2D materials. However, the design and preparation of high‐performance metal catalysts for AOR with 2D materials as supports still have a long way to reach due to the immature and imperfect preparation and characterization techniques at this stage.

**Figure 1:**
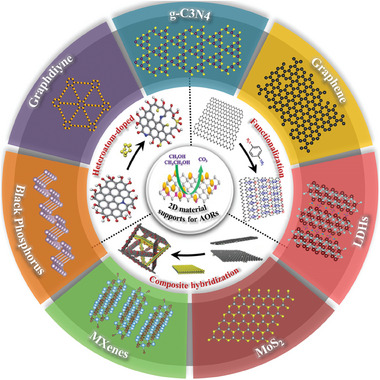
Schematic representation of the fundamental structure and modification strategies of 2D materials support for AOR.

More recently, various 2D nanomaterials with layered structures were extensively utilized as support materials for the preparation of high‐performance supported metal catalysts, such as graphene, graphitic carbon nitride (g‐C_3_N_4_), MXenes (metal carbides and nitrides), graphdiyne (GDY), and molybdenum disulfide (MoS_2_).^[^ ^18^, ^19^, ^20^, ^21^ ^]^ As an emerging area, novel supported metal electrocatalysts for AORs have been developed by tuning the structure and/or morphology of 2D layer material supports with modification strategies.^[^ ^22^, ^23^ ^]^ Unfortunately, there is little systematic and overall design guidance for supported metal catalysts for AORs from the perspective of 2D support materials. This stimulated us to write this review article valuating the recent progress in the exploration of 2D layer material as catalyst supports for AORs, with emphasis on the modification strategies, such as surface molecular functionalization, heteroatom doping, and composite hybridization (**Figure** **1**). Meanwhile, the developments, tendencies, and existing issues are discussed in detail based on the reported studies. This will promote further efforts to developing highly efficient electrocatalysts for AORs.

## 2 Principles of Alcohol Oxidation Reaction

### 2.1 Catalytic Mechanism of Alcohol Oxidation Reaction

DAFCs are an electrochemical conversion device, in which the oxidation of alcohols or their aqueous solutions takes place on the anode of the fuel cell to produce electrical energy.^[^ ^24^ ^]^ However, its overall device performance and efficiency are significantly limited by the difficult activation of AORs. In the recent years, remarkable developments have been achieved in the field. The alcohols that have been employed in DAFCs are C1 alcohols (such as methanol) and C2 alcohols (such as ethanol and ethylene glycol). **Table** **1** summarizes the relevant thermodynamic data for the complete electrochemical oxidation of several typical low‐carbon alcohols. Since the carbon number of alcohols has remarkable influences on electrochemical oxidation, a brief introduction of the AOR mechanism is presented in the following subsections using methanol and ethanol as examples.

**Table 1:** Relevant thermodynamic data for the complete electrochemical oxidation of some typical low‐carbon alcohols. Reproduced with permission.^[^ ^25^ ^]^ Copyright 2002, Elsevier.

Fuel	Δ*G^θ^* [kJ mol^−1^]	E*θ cell* [V]	*W_e_* [kW h kg^−1^]	Δ*H^θ^* [kJ mol^−1^]	*ε_r_*
Methanol	‐702	1.213	6.09	‐726	0.967
Ethanol	‐1325	1.145	8.00	‐1367	0.969
*n‐*Propanol	‐1853	1.067	8.58	‐2021	0.916
Butanol	‐2381	1.029	8.93	‐2676	0.890

#### 2.1.1 Oxidation Mechanism of Methanol

Methanol is the earliest and most widely used fuel for DAFC due to its simple molecular structure, absence of C‐C bonds, ease of oxidation, and high energy density. The mechanism of anodic electro‐oxidation of alcohols is generally complex. In the anode, methanol molecule has an oxidation reaction in the effect of the catalyst, which completely oxidizes to form CO_2_ and releases 6e^−^ at simultaneously. The complete methanol oxidation reactions (MORs) in acidic and alkaline solutions can be expressed as follows

(1) $${\mathrm{CH}}_{3}\mathrm{OH}\;+\;H_{2}O\;\to {\;{\mathrm{CO}}_{2}\;+\;6H^{+}\;+\;6e}^{-}\;\;(\mathrm{acidic}\;\mathrm{medium})$$

(2) $${{\mathrm{CH}}_{3}\mathrm{OH}\;\;+\;\;6\mathrm{OH}}^{-}\;\to \;{\;{\mathrm{CO}}_{2}\;\;+\;\;5H_{2}O\;\;+\;\;6e}^{-}\;(\mathrm{alkaline}\;\mathrm{medium})$$

**Figure 2:**
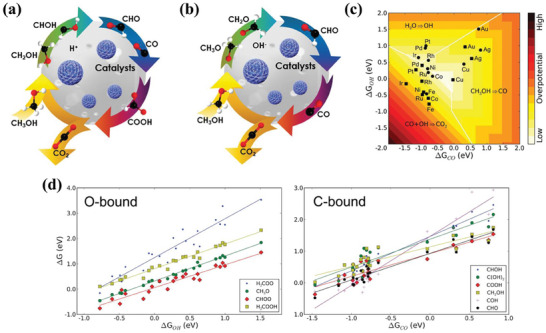
Mechanisms of MOR on the catalysts in a) acidic media and b) alkaline media. Reproduced with permission.^[^ ^26^ ^]^ Copyright 2021, Wiley‐VCH. c) Volcano plot for the indirect path, for stepped surfaces (squares) and flat surfaces (circles). The overpotential minimizes over an area (white) of optimal CO and OH adsorption energies. d) Linear relationships between the adsorption free energies of MOR intermediates and the adsorption energy of OH (left) or CO (right), for O‐ or C‐bound species, respectively. Reproduced with permission.^[^ ^29^ ^]^ Copyright 2012, American Chemical Society.

The mechanism of MOR in acidic and alkaline solutions is shown in **Figure** **2**a,b. The MOR process is generally considered to consist of four elementary steps. First, methanol is adsorbed on the surface catalysts. In alkaline solution, OH^−^ is adsorbed on the surface as well. Then, methanol dehydrogenation is accompanied by the oxidation of the C─H and O─H bonds to produce various carbonaceous intermediates hydroxymethyl CH_2_OH_ads_ (acidic medium) or methoxyl CH_3_O_ads_ (alkaline medium), CH_2_O_ads_, CHO_ads_ (alkaline medium) and ultimately, stable intermediates CO_ads_. Further, H_2_O is adsorbed onto the surface of the catalyst and activated to form OH_ads_ and OH^−^ (alkaline medium). Finally, the CO_ads_ intermediate is oxidized by OH_ads_ and OH^−^ (alkaline medium) to form CO_2_ gas. The formation of stable carbonaceous intermediates affects the efficiency and kinetics of MOR. Among them, the difficult desorption of CO will inhibit the further adsorption of methanol, leading to a decrease in the reaction activity. Therefore, it is crucial to generate OH_ads_ to effectively remove poisonous carbonaceous intermediates from this reaction. It is widely accepted that the MOR performance is better in alkaline electrolytes than in acidic electrolytes because the high concentration of OH^−^ is beneficial for removing the toxic carbonaceous intermediates CO_ads_.^[^ ^26^ ^]^ Alternatively, in the direct pathway, the key intermediate CHO_ads_ are converted to HCOOH_ads_ and then oxidized to CO_2_ without the formation of poisoning intermediates CO.^[^ ^27^ ^]^ Currently, the MOR mechanisms are mainly proposed based on the precious metal electrocatalysts, such as Pt and Pd.^[^ ^28^ ^]^

The two surface reactivity parameters, CO binding energy (ΔG_CO_) and OH binding energy (ΔG_OH_) were plotted as a 2D‐filled contour map against the MOR potential of U_MOR_^i^ as shown in Figure 2c. The plot hints at conditions to be satisfied for enhanced catalysis (e.g., optimal surface reactivity). A framework was proposed to evaluate MOR based on two reactivity parameters, the free energy of adsorption of CO_ads_ and OH_ads_ on the surfaces. As shown in Figure 2d, one can see that the binding energy of other intermediates is proportional to the binding energy of these two intermediates.^[^ ^29^ ^]^ Two sets of linear relationships could be established for the states of the C‐bound species and the O‐bound species. Therefore, an excellent catalyst for MOR requires three main capabilities: methanol dehydrogenation, water activation for OH species formation, and the removal of CO_ads_ and other intermediates from the surface.

#### 2.1.2 Oxidation Mechanism of Ethanol

Compared with methanol, ethanol as a fuel has the advantages of safety, non‐toxicity, and higher energy density (8.00 kW h kg^−1^).^[^ ^30^, ^31^ ^]^ Due to the presence of a C─C bond, its complete electrochemical oxidation to CO_2_ and H_2_O is very difficult under conventional conditions. The ethanol oxidation reaction (EOR) mechanism is more complex than that of MOR, including complex parallel and consecutive reactions. Two pathways were proposed. One is the C1 pathway including the complete oxidation of ethanol as a 12‐electron transfer process with the C─C bond cleavage. The other is the C2 pathway without the breaking of the C─C bond, in which ethanol is converted to formaldehyde in the 2‐electron dehydrogenation process and to acetic acid in the 4‐electron transfer process. The oxidation of ethanol to form acetaldehyde, acetic acid, or CO_2_ can be expressed as follows

(3) $$C_{2}H_{5}\mathrm{OH}\;\to {\;{\mathrm{CH}}_{3}\mathrm{CHO}\;+\;2H^{+}\;+\;2e}^{-}$$

(4) $$C_{2}H_{5}\mathrm{OH}\;+\;H_{2}O\;\to {\;{\mathrm{CH}}_{3}\mathrm{COOH}\;+\;4H^{+}\;+\;4e}^{-}$$

(5) $${\mathrm{CH}}_{3}{\mathrm{CH}}_{2}\mathrm{OH}\;+\;3H_{2}O\;\to {\;2{\mathrm{CO}}_{2}\;+\;12H^{+}\;+\;12e}^{-}$$

The research results show that the EOR occurs mainly as the 4‐electron process, with acetaldehyde and acetic acid as the main by‐products via several main steps. First, ethanol is adsorbed on the surface of the catalysts. In alkaline solution, OH^−^ is adsorbed on the surface as well. Then, ethanol dissociates, accompanied by the cleavage of the C─H and O─H bond, producing various carbonaceous intermediates CH_3_CH_2_O_ads_, CH_3_CHO, and CH_3_CO_ads_. Furthermore, H_2_O is adsorbed onto the surface of the catalyst and activated to form OH_ads_ and OH^−^ (alkaline medium). The CH_3_CH_2_O_ads_ and CH_3_CO_ads_ produced by C─H and O─H bond oxidation process are oxidized by OH_ads_ to generate CH_3_COOH. In the presence of a higher‐performance catalyst, the intermediate CH_3_CO_ads_ further converts CO_ads_ because it involves the breaking of the C─C bond. In its electrooxidation reaction, the formation of stable carbonaceous intermediates is still a key issue that seriously hinders the progress of the reaction.

There are few reports on the oxidation mechanisms of other high‐carbon alcohols.^[^ ^32^, ^33^ ^]^ Their complete oxidation to CO_2_ and H_2_O has not yet been achieved, leading to mixed products. Moreover, the oxidation of alcohol molecules is very difficult due to the hydrocarbon group (e.g., methyl, ethyl). Therefore, the higher activity of catalysts is required to oxidize alcohols with the longer hydrocarbon groups.

### 2.2 Catalyst Design for Microstructure and Electronic Structure

The catalytic activity of materials depends on their micro‐morphology and electronic structure, promoting recent attempts to explore supported metal catalysts. It is well known that reducing the size of catalysts would increase the number of exposed active sites and lead to improved MOR performance. The 2D materials support can effectively disperse and anchor the active metal NPs, thus increasing the number of active sites of the catalyst to improve the catalytic activity; it can also prevent the agglomeration of the metal NPs during the catalytic reaction and improve the durability of the catalyst. Additionally, the interaction between the carrier and the metal plays a crucial role in the activity and stability of the catalyst. The modulation of electronic structure between the support and metal is crucial in designing efficient supported metal catalysts, which has become a prominent research topic in recent years.

As early as the 1930s, G.M. Schwab proposed the concept of “electronic factor” to describe how electronic interactions influence the catalytic behavior of supported catalysts.^[^ ^34^ ^]^ The consideration of electron transfer between the metal and support was initially focused on catalysis but later expanded to encompass interactions between any metallic species and the support. In 2012, C.T. Campbell proposed the concept of electronic metal‐support interaction (EMSI), which suggests that the metal‐support interaction induces electronic perturbation on the metal, as reflected by its density of states (DOS).^[^ ^35^ ^]^ The alteration of the DOS induces changes in both the Fermi energy level and the d‐band center. On an active metal surface, modifications in the d‐orbitals can influence its binding energy with reacting intermediates, thereby impacting their adsorption, activation, and desorption behavior.

**Figure 3:**
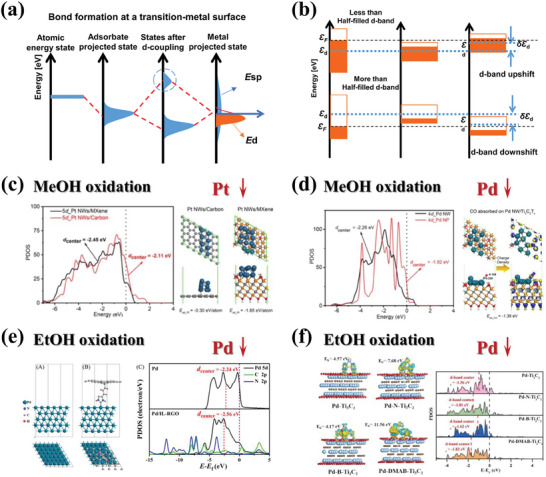
a) Schematic illustration of the interaction between the absorbates and the transition metal surface. b) A schematic illustration of the d‐band center position for the early transition metals and late transition metals. c) *d*‐PDOS plots of Pt NWs/Carbon and Pt NWs/MXene models; Relaxed atomic structures of Pt NWs/Carbon and Pt NWs/MXene models. Reproduced with permission.^[^ ^38^ ^]^ Copyright 2022, Elsevier. d) The *d*‐PDOS plots of Pd NP and Pd NW on Ti_3_C_2_T_x_; The atomic models for CO molecules adsorbed on Pd NW/Ti_3_C_2_T_x_. Reproduced with permission.^[^ ^39^ ^]^ Copyright 2022, Elsevier. e) Optimized atomic structures of Pd and Pd/IL‐RGO; Projected DOS for Pd and Pd/IL‐RGO system. Reproduced with permission.^[^ ^40^ ^]^ Copyright 2021, American Chemical Society. f) The binding energy; d‐band center of Pd‐Ti_3_C_2_, Pd‐N‐Ti_3_C_2_, Pd‐B‐Ti_3_C_2_, and Pd‐DMAB‐Ti_3_C_2_. Reproduced with permission.^[^ ^41^ ^]^ Copyright 2023, Elsevier.

The d‐band center theory, proposed by Nørskov, has been successfully utilized to elucidate and predict the activity changes of 3d metals in various reactions.^[^ ^36^ ^]^ For transition metals, as illustrated in **Figure** **3**a, their electronic states can be categorized into sp‐band and d‐band originating from valence s and p atomic orbitals with broad overlapping shapes and localized valence d orbitals, respectively. When small molecules approach the surface, their coupling to the transition metal can be considered a two‐step process involving coupling to both surface sp‐states and d‐states (Figure 3a). The assumption of independent ΔE_sp_ for simple transition metals makes the similar sp‐band coupling quite reasonable, thereby implying a direct relationship between adsorption energy for adsorbates and d‐band electronic states. In the simplest description of the d‐band theory, the descriptor for d‐band electrons is represented by the d‐band center (Ɛ_d_). Consequently, a stronger interaction can be expected if Ɛ_d_ shifts toward the Fermi level, while a weaker interaction accompanies an Ɛ_d_ shift away from the Fermi level. Specifically, for transition metals with more than half‐filled d orbitals, the Ɛ_d_ shifts upward to maintain the degree of filling when the bandwidth becomes narrower. Conversely, for those with less than half‐filled d orbitals, a downward shift of Ɛ_d_ is required to maintain the number of electrons in the d‐band (Figure 3b). The shift in d‐band centers can significantly impact the adsorption energy of intermediates and potentially alter reaction pathways and energy barriers at rate‐determining steps (RDS) and thus change reaction kinetics during catalytic processes.^[^ ^37^ ^]^ Due to the significance of Ɛ_d_ modulation in optimizing electrocatalytic processes, there have been tremendous efforts devoted to finding cost‐effective large‐scale and highly efficient strategies that can effectively change the d‐band center positions of transition metals. In this review, we specifically focus on the impact of the d‐band center on the electrooxidation performance of alcohols (methanol, ethanol, etc.) using noble metals (Pt, Pd) supported by 2D materials. Additionally, the reference catalyst support is also included for the discussion.

#### 2.2.1 Methanol Oxidation

Pt NWs/Ti_3_C_2_T_x_ (*Pt: downshift of Ɛ_d_* vs Pt NWs/C)^[^ ^38^ ^]^: By coupling 1D grain boundary‐rich Pt nanowires (NWs) with ultrathin Ti_3_C_2_T_x_ nanosheets, Huang et al. prepared the heterojunction Pt NWs/MXene catalyst to evaluate the methanol oxidation performance in the acid media. The heterojunction supported metal catalyst exhibits a series of structural merits, such as large surface area, numerous grain boundary sites, ameliorative electronic structure, and excellent electron conductivity. Density functional theory (DFT) calculations have validated a downshift d‐band center and a much stronger electronic interaction of Pt NWs grown on MXene than the carbon matrix. A large electrochemically active surface area (ECSA) (105.5 m^2^ g^−1^), mass activity (1621.5 mA mg^−1^), as well as superior long‐term durability, make Pt NWs/MXene more competitive than conventional Pt NP/carbon catalysts with the same Pt loading (Figure 3c).

*Pd NWs/Ti_3_C_2_ (Pd: downshift of Ɛ_d_ vs Pd NPs/Ti_3_C_2_)* ^[^ ^39^ ^]^: Huang et al. constructed the quasi‐1D worm‐shaped Pd nanocrystals strongly coupled with positively‐charged polyelectrolyte‐modified Ti_3_C_2_T_x_ via the direct electrostatic attractions. The 1D/2D heterointerfaces induce significant electron transfer from the Pd NWs to the Ti_3_C_2_, and a reduced d‐band center can be observed (Figure 3d). The downshift of the d‐band center causes a lower CO adsorption energy of the Pd NWs/Ti_3_C_2_ (−1.38 eV) than that for Pd NPs/Ti_3_C_2_ (−1.95 eV). The CO molecule loses electrons of 0.166 and 0.040 *e* on Pd NP/Ti_3_C_2_T_x_ and Pd NW/Ti_3_C_2_T_x_, respectively, proving again the weaker adsorption of CO molecule on the worm‐shaped Pd configuration. The as‐fabricated Pd NWs/PDDA‐MX hybrid shows superior electrocatalytic performance with a large ECSA of 105.3 m^2^ g^−1^, a high mass activity of 1526.5 mA mg^−1^, and reliable long‐term durability toward alkaline methanol oxidation.

#### 2.2.2 Ethanol Oxidation

*Pd/IL‐RGO (Pd: downshift of Ɛ_d_ vs Pd)* ^[^ ^40^ ^]^: Wu et al. designed and prepared the Pd/IL‐RGO with a 3D flower‐like structure by a universal template‐free approach followed by an in situ classical wet chemical growth method. DFT calculations disclose that the dramatically enhanced catalytic performance is ascribed to the strong electronic interaction between Pd and the ionic liquid (IL), which generates a downshift of the d‐band center for Pd atoms and thereby promotes the durability toward CO‐like intermediates and the electrocatalytic reaction kinetics (Figure 3e). Pd/IL‐RGO presents enhanced EOR electrocatalytic activities and stabilities compared with Pd/RGO and commercial Pd/C catalysts.

*Pd/DMAB‐Ti_3_C_2_ (Pd: downshift of Ɛ_d_ vs Pd/Ti_3_C_2_, Pd/N‐Ti_3_C_2_, Pd/B‐Ti_3_C_2_)* ^[^ ^41^ ^]^: our group prepared a novel B, N co‐doped Ti_3_C_2_ supported Pd catalyst by uses DMAB as a heteroatom dopant and a metal‐reducing agent at the same time through a rapid one‐step hydrothermal method. The Pd/DMAB‐Ti_3_C_2_ exhibited high electrocatalytic activity and stability for EOR. It has been proved, experimentally and theoretically, that cooping MXene with B and N heteroatoms can effectively increase the interlayer spacing of MXene and optimize the distribution of metal Pd, which enhances the electronic interaction of metal Pd and downshift the d‐band center, thus regulating the selective adsorption of EOR intermediates (Figure 3f).

As discussed above, the shift of the d‐band center provides a qualitative explanation for the modulation of the binding affinity of key intermediates on electrocatalyst surfaces. However, limited results were reported on an algebraic description of binding parameters associated with catalytic performance, despite the common provision of d‐band center position variations. This discrepancy may arise from differences in calculation methods and input parameters used to determine the positions of the d‐band center. Nevertheless, a precise algebraic expression of the relationship between the binding parameters and the intrinsic activity of electrocatalysts would further favor the rational design of the 2D materials‐supported metal electrocatalysts with desired activity and stability.

## 3 Modification Strategies for 2D Materials as Electroatalyst Supports

**Figure 4:**
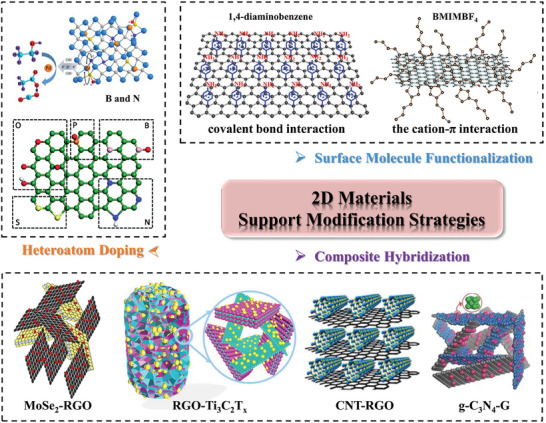
Schematic representation of modification strategies for 2D materials support. The diagram of surface molecule functionalization is Reproduced with permission.^[^ ^42^, ^43^ ^]^ Copyright 2019, American Chemical Society; Copyright 2016, The Royal Society of Chemistry. The diagram of heteroatom doping is Reproduced with permission.^[^ ^44^, ^45^ ^]^ Copyright 2015, The Royal Society of Chemistry; Copyright 2022, American Chemical Society. The diagram of composite hybridization is Reproduced with permission.^[^ ^46^, ^47^, ^48^, ^49^ ^]^ Copyright 2023, Elsevier; Copyright 2019, American Chemical Society; Copyright 2015, Springer Nature; Copyright 2014, Wiley‐VCH.

As mentioned above, the particle size, dispersion, and especially the electronic structure (d‐band centers) of the active metal in the supported‐metal catalyst determine its catalytic activity. The modification strategy of the support can often change its surface properties and electronic structure, which can significantly affect the microscopic morphology and d‐band centers of the loaded metal, thus altering the properties and catalytic activity of the catalyst. Furthermore, the strong interaction between the metal and the support also improves the stability of the catalyst. In this section, modification strategies for 2D support that can be employed to enhance the catalytic activity of loaded metals are summarized (**Figure** **4**), including surface molecule functionalization (non‐covalent interactions and covalent interactions), heteroatom doping, and composite hybridization.

### 3.1 Surface Molecule Functionalization Strategy

Surface molecular functionalization strategies refer to introducing functional molecular groups on the surface of 2D materials, which can have significant influences on their properties and performance, such as hydrophilic nature, electronic structure, and stability, thus adjusting the surface structure and electronic structure of the loaded active metal. Also, these surface groups affect the loading of the active metal and further influence the catalytic performance of the supported‐metal catalysts. The process is relatively milder compared to the harsh conditions of other engineering strategies and has been widely used to enhance the loading performance of 2D support. Furthermore, it is the most effective strategy for dispersing metal NPs and controlling particle size. According to the type of molecules and 2D material supports, the surface molecular functionalization types can be classified into covalent and non‐covalent interactions.

#### 3.1.1 Covalent Interactions

The preparation of 2D materials usually involves the inevitable introduction of surface groups, which also provides a platform for surface molecular functionalization through covalent strategies. In this strategy, the functional molecular groups are connected to the 2D materials through the form of covalent bonds, which are usually realized by effective chemical reactions. Due to the presence of covalent bonds, the functional molecules can be firmly grafted onto the 2D material support at one end, while the other end can be used as an anchor site for the active metal NPs to prevent them from aggregating during the reaction process. The hydrophilicity of the 2D support can also be altered to provide better dispersion and more available specific surface area. Among the various functionalization groups, surface modification of 2D support by functional organic molecules is an attractive approach. Due to their ability to serve as attachment sites for the growth of active metal NPs, some organic small molecules rich in amino and carboxyl groups are widely used to modify 2D materials. Moreover, it has been shown that the metal‐organic interface interactions generated between the functional groups of organic molecules and metal NPs can effectively tune the surface electronic structure of the metal, thus improving its electrocatalytic activity and stability.^[^ ^50^ ^]^

#### 3.1.2 Non‐Covalent Interactions

2D materials can be modified by functional molecular through typical interactions, including Vander Waals force, π‐π stacking interactions, hydrogen bonding, or electrostatic interactions. This strategy is simpler compared to the covalent strategy. Compared to covalent strategies, this strategy has more flexible requirements for 2D substrate materials and functional molecules, and the preparation process is relatively simple. Commonly used functional molecules include organic small molecules, ILs, and conductive polymers. For instance, reduced graphene oxide (rGO) can be modified by polyaniline (PANI) through a variety of non‐covalent interactions, including *π*–*π* interactions, hydrogen bonding, and electrostatic interactions.^[^ ^51^ ^]^ Moreover, the magnitude of non‐covalent interactions, such as *π*–*π* interactions, can also be modulated by changing the size of the conjugated aromatic groups of the functional molecule.^[^ ^52^ ^]^

### 3.2 Heteroatom Doping Strategy

Introducing additional species into the 2D materials structures, e.g., heteroatom doping, is an efficient way to engineer catalyst support with regulated electronic structures and unique properties.^[^ ^53^ ^]^ The heteroatoms cause a change in the DOS on the surface of the 2D support and thus the variation of work functions.^[^ ^23^ ^]^ Heteroatom‐doped 2D material support will produce not only more metal anchor sites but also strong metal‐support interactions with the loaded metal. This strong interaction induces electron transfer between the metal and the support, which changes the electronic structure and d‐band center of the active metal and improves the catalytic activity. For example, Nicolas Alonso‐Vante's group investigated the EMSI between N‐doped graphene and Pt NPs.^[^ ^54^ ^]^ The results show that the doped N atoms form Pt‐N chemical bonds with the surface deposited Pt NPs. This interaction allows for an electronic transfer from Pt to the support, which alters the electronic structure of the active metal and further induces changes in its surface adsorption energy, thus enhancing the activity of the catalyst for the reaction. Furthermore, the presence of this strong interaction can effectively prevent the metal NPs from detachment and agglomeration from the 2D support during the catalytic reaction. To date, experiments and theoretical calculations have shown that heteroatom doping can effectively change the electronic structure and catalytic activity of various 2D supported‐metal catalysts such as graphene and MXene. The type of heteroatoms can be categorized into non‐metal‐atom element doping and metal‐atom doping. The number of elemental species can be divided into single elemental doping and co‐doping. Considering the atomic radius and the ease of doping, the main heteroatoms currently being used in 2D materials are N, B, S, Se, P, etc. For instance, the widely reported N‐, B‐, and S‐doped graphene as well as N‐ and B‐doped MXene.^[^ ^55^, ^56^ ^]^ Different types of heteroatoms and their different configurations have remarkable effects on the performance of the supported metal catalysts. The classic example is that for oxygen reduction reaction (ORR) catalysis, graphitic‐N and pyridinic‐N are the key sites for N‐doped graphene nanomaterials.^[^ ^57^ ^]^

In addition to heteroatom doping, the approaches to construct defects in 2D support materials include the introduction of vacancy defects, topological defects, and so on.^[^ ^58^ ^]^ However, these types of defects in 2D materials are greatly influenced by the material structure and synthesis, and their characterization is difficult. The approach of constructing defects such as oxygen vacancy on 2D supports as anchor sites for active metal NPs has rarely been reported in the field of AORs. Therefore, the inherent defects in 2D support materials are briefly discussed and introduced as their structural features in this review, such as oxygen vacancy in graphene and edge defects in MXenes.

### 3.3 Composite Hybridization Strategy

Composite hybridization of 2D materials is another important approach for designing high‐efficient electrocatalyst supports. Due to van der Waals forces and *π*–*π* interactions, it is inevitable that 2D materials support irreversibly agglomerate during the catalytic process, leading to the reduction of the specific surface area and blockage of the pores. To solve these problems, composites are prepared by introducing a “spacer”. As a result, 2D‐based hybrid support will show desirable properties for electrocatalytic applications, including porous structure, large surface area, abundant active sites, and fast electron transfer. The combinations of 2D material hybridization composites are diverse. As an example, the classical 2D material graphene can be hybridized with 1D carbon nanotubes (CNTs) or multi‐walled carbon nanotubes (NWCNTs), and it can also be composited with other 2D materials such as MoS_2_ and MXene to prepare high‐performance catalyst support.^[^ ^59^ ^]^ Thus, the composite hybridization strategies for 2D materials offer the possibility of exploring low‐cost, high‐activity supported‐metal catalysts for AORs.

## 4 2D Materials as Electrocatalyst Supports for AORs

Supported metal catalysts are extensively used in AORs due to the merits of recycling and high atomic usage. The individually dispersed metal NPs can be prevented from aggregating, and their stability is maintained during the reaction by appropriate metal‐support binding. Furthermore, the interaction between the metal and the support also influences the catalytic properties of the metal NPs.^[^ ^60^ ^]^ The type of support material significantly affects the morphology and electronic structure of metal NPs. Compared to substrates of 1D and 3D structures, 2D support materials, especially thin atomic layers, more easily expose active metal NPs to the reactants, effectively increasing the utilization of active metals and maximizing the electrocatalytic reaction rate as well.

More recently, AOR electrocatalysts based on 2D material supports have attracted much attention.^[^ ^61^, ^62^ ^]^ There are many advantages that make 2D materials the most promising support for metal catalysts. First, 2D materials have a very large surface area, which can provide a sufficient number of anchoring sites for metal NPs and fully expose them to the reactants. Second, 2D materials exhibit shorter charge migration distances and 2D charge transport channels. Third, compared to 1D and 3D structures, 2D materials are more easily tuned by various means for their binding to supported metal NPs due to uniformly exposed single layers and smaller electronic DOS. To date, hundreds of 2D materials that can exist stably, such as graphene, MXenes, g‐C_3_N_4_, GDY, and MoS_2_ have been invented.^[^ ^63^, ^64^ ^]^ Most of them can be stripped from their bulk weight to form single or multilayer materials. The interaction between 2D material supports and metals can be modulated by doping, chemical functionalization, or hybridization. Impressive advances have been achieved in improving electrocatalytic activity, stability, and selectivity through the rational design of 2D supports. In the following sections, we will focus on these 2D material supports, optimization strategies, and underlying mechanisms.

### 4.1 Graphene

Graphene, as a highly representative 2D material, opens a new era in the research field of low‐dimensional materials. Since its successful exfoliation in 2004, its unique planar structure and novel electronic properties have attracted extensive attention, and it is one of the most promising and widely used materials in the field of electrocatalysis.^[^ ^65^, ^66^, ^67^, ^68^, ^69^ ^]^ As is known to all, graphene is a 2D single‐layer sheet of sp^2^ hybridized carbon atoms with a hexagonal packed structure.^[^ ^70^ ^]^ In comparison with CNTs and carbon nanofibers (CNFs), graphene possesses not only similar stable physical properties but also larger theoretical surface areas (2630 m^2^ g^−1^), which makes it an excellent support material for metal NPs. In addition to pristine graphene, there is also graphene oxide (GO) and rGO, all of which have been realized production on a large scale. Up until now, several approaches have been reported for the fabrication or synthesis of graphene and its derivatives.^[^ ^71^ ^]^ Currently, the Hummers method is the most popular way of graphene preparation, which uses graphite powder as the raw material to prepare GO by oxidative exfoliation and rGO by chemical reduction.^[^ ^72^ ^]^ GO or rGO surfaces prepared using this approach will be abundant functional groups and defects. These defects will act as anchoring points for the metal NPs and may induce some modulation of the morphology and electronic structure of the loaded metal NPs, which will enhance the catalytic activity for AORs.

**Figure 5:**
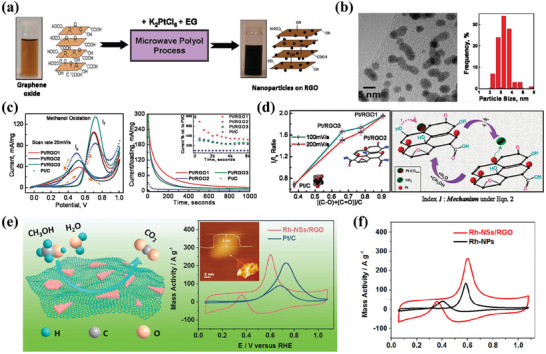
a) Scheme of MWAPP synthesis of Pt/RGO hybrids. b) TEM image and Pt NPs size distribution on Pt/RGO. c) CV responses of all samples. d) Dependence of *I*_f_/*I*_b_ ratio on contribution of residual oxygen species, and Schematic diagram explaining the conversion of adsorbed CO_ads_ species to CO_2_ on Pt/RGO hybrids. Reproduced with permission.^[^ ^75^ ^]^ Copyright 2010, American Chemical Society. e) Scheme of Rh‐NSs/RGO hybrids and ECSA‐normalized CVs of different catalysts. f) Rh mass‐normalized CVs of the Rh‐NSs hybrids and Rh‐NSs/RGO hybrids. Reproduced with permission.^[^ ^77^ ^]^ Copyright 2017, American Chemical Society.

Further, Ramasubramaniam et al. showed by combining DFT and bond‐order potential calculations that the formation of metal‐carbon bonds at graphene defects affects the average length of the bond, which influences the strain of the metal clusters and consequently produces stronger bonding.^[^ ^73^ ^]^ As a result, the charge transfer from the metal clusters to the substrate is increased, while the d‐band center of the metal clusters is substantially shifted downward, which provides a possible pathway to enhance the stability and CO tolerance of Pt NPs on graphene. This result provides a theoretical basis for the design of high‐performance graphene‐based supported metal catalysts for AORs. Compared to other carbon materials such as carbon black (CB), CNTs, and MWCNTs, graphene is an excellent support for increasing the catalytic performance of metal NPs for AORs. For instance, the catalyst Pt/GNS prepared with rGO nanosheets as the support exhibited higher efficiency for MOR than Pt/CB, while the CO adsorption rate was 40 times slower than those of Pt/CB.^[^ ^74^ ^]^ It was attributed to the strong interactions between the defect sites on GNS and Pt atoms, resulting in a decrease in the particle size of the Pt NPs and a change in the metal dispersion. Furthermore, the presence of residual oxygen groups on rGO plays a vital role in the removal of carbonaceous species from the adjacent Pt sites (**Figure** **5**a–c).^[^ ^75^ ^]^ The Pt/rGO catalyst showed excellent electrocatalytic activity and CO poisoning tolerance for MOR, superior to the commercial Pt/C electrocatalysts (Figure 5d). The microstructure of the graphene support material also affects the electrochemical performance of the metal catalyst. For example, compared with graphene nanosheets (GNTs) with flat surfaces, the rippled morphology is more suitable as support for metal NPs because the rippled surface is beneficial for obtaining small metal NPs with highly uniform particle distribution.^[^ ^76^ ^]^

The strategy of using graphene as a support to enhance the catalytic performance of metal catalysts has gained wide popularity in the research of AORs.^[^ ^78^ ^]^ In addition to the most commonly used precious metal Pt and Pd, some other metals with alcohol catalytic activity, such as Ni, Rh, Au, etc., have been loaded on graphene to prepare supported metal catalysts.^[^ ^79^ ^]^ The prepared Rh‐NSs/RGO catalysts exhibited great electrocatalytic activity for MOR in an alkaline medium, which is much better than that of single‐component Rh NPs (Figure 5e,f).^[^ ^77^ ^]^ Similarly, the performance of alloy catalysts with graphene as support could also be realized to be substantially improved.^[^ ^80^, ^81^ ^]^ For instance, in comparison to CB as catalyst support, graphene can more effectively enhance the electrocatalytic activity of Pt‐Ru NPs for the oxidation of methanol and ethanol into CO_2_.^[^ ^82^ ^]^

In addition to direct metal loading, the large number of functional groups on the surface of graphene (GO or rGO) provides the opportunity for further modification. In recent research, a series of high‐performance catalysts for AORs have been produced by improving the dispersion of metal NPs and enhancing the interaction between support and metal NPs through strategies such as surface molecules functionalization, heteroatom doping, and composite hybridization. They are discussed in detail in the following sections. **Table** **2** summarizes the various metal graphene‐based electrocatalysts used in the AOR.

**Table 2:** Summary of the graphene‐based metal catalysts for the AOR.

Catalyst	Electrolyte	ECSA [m^2^ g^−1^]	Mass activity [mA mg^−1^]	Specific activity [mA cm^−2^]	Stability (Retention rate)	Refs.
Rh/rGO	1.0 M CH_3_OH + 1.0 M KOH	48.66	264	‐	‐	[77]
Pd/G	0.5 M CH_3_OH + 1.0 M NaOH	81.60	‐	1.858	93.4% after 100 cycles	[83]
PtRu/rGO	1.0 M CH_3_OH + 0.5 M H_2_SO_4_	130.46	570	1.1	‐	[84]
PtPd/rGO	1.0 M CH_3_OH + 0.1 M HClO_4_	197	198	1.31	94.4% after 1000 cycles	[85]
PtNi/G	1.0 M CH_3_OH + 0.5 M NaOH	37.6	100.8	‐	76.3% after 900 cycles	[86]
PtCu/AP‐G	0.5 M CH_3_OH + 0.1 M NaOH	91.89	3610	‐	86.37% after 1500 cycles	[87]
Pd‐CeO_2‐x_/F‐G	1.0 M C_2_H_5_OH + 1.0 M KOH	37.7	2655.8	7.04	22.6% after 500 cycles	[88]
Pd/DPHE‐G	1.0 M CH_3_OH + 1.0 M KOH	54.8	1539.0	2.81	23.7% after 500 cycles	[89]
	1.0 M C_2_H_5_OH + 1.0 M KOH		2464.8	4.50	18.9% after 500 cycles	
Pd/IL‐rGO	1.0 M C_2_H_5_OH + 1.0 M KOH	26.8	2552.1	9.52	67.0% after 500 cycles	[40]
Pt_3_Ni/PPy‐G	1.0 M C_2_H_5_OH + 1.0 M KOH	44.7	679.7	‐	80% after 300 cycles	[90]
Pd‐PdO_x_/NH_2_‐G	1.0 M C_2_H_5_OH + 1.0 M KOH	24.9	1319.9	5.30	33.1% after 500 cycles	[42]
PdP/PDA‐G	1.0 M C_2_H_5_OH + 1.0 M KOH	63.3	1733.2	‐	43.5% after 500 cycles	[91]
PtAg/N‐G	1.0 M C_2_H_5_OH + 1.0 M KOH	41.09	3598.4	8.76	49.8% after 500 cycles	[92]
PtNiP/P‐G	1.0 M CH_3_OH + 0.5 M H_2_SO_4_	126.5	826.1	0.65	64% after 1000 cycles	[93]
Pt/N‐G	2.0 M CH_3_OH + 1.0 M H_2_SO_4_	60.9	1283.1		‐	[94]
Pd/NS‐G	1.0 M CH_3_OH + 0.5 M NaOH	103.6	399.3	11.3	41.6% after 500 cycles	[95]
Pt/rGO‐Ti_3_C_2_T_x_	0.5 M CH_3_OH + 0.5 M H_2_SO_4_	90.1	1102	1.2	24.4% after 1000 cycles	[47]
Pd/Ni(OH)_2_‐rGO	1.0 M C_2_H_5_OH + 1.0 M KOH	40.3	1546	‐	90% after 40000 cycles	[96]
Pt/CNT‐rGO	1.0 M CH_3_OH + 1.0 M H_2_SO_4_	85.21	702.4	0.82	81.5% after 100 cycles	[48]
Pd/PEDOT‐G	1.0 M C_2_H_5_OH + 1.0 M KOH	13.2	458.5	‐	90.9% after 300 cycles	[97]

#### 4.1.1 Surface Molecule Functionalization

Graphene (GO and rGO) is considered to be an excellent support for metal NPs due to rich oxygen‐containing functional groups such as hydroxyl, epoxy, keto, and carboxyl groups on its surface. This also opens up the possibility of its functionalization.^[^ ^42^ ^]^ The activity of the catalyst can be enhanced by attaching functional groups to the graphene support that can influence the nucleation of the metal particles to enhance and change the electronic structure of the catalyst, while the durability is augmented due to the enhanced chemical binding energy between the metal particles and the support. In recent years, many related studies have been continuously carried out.

**Figure 6:**
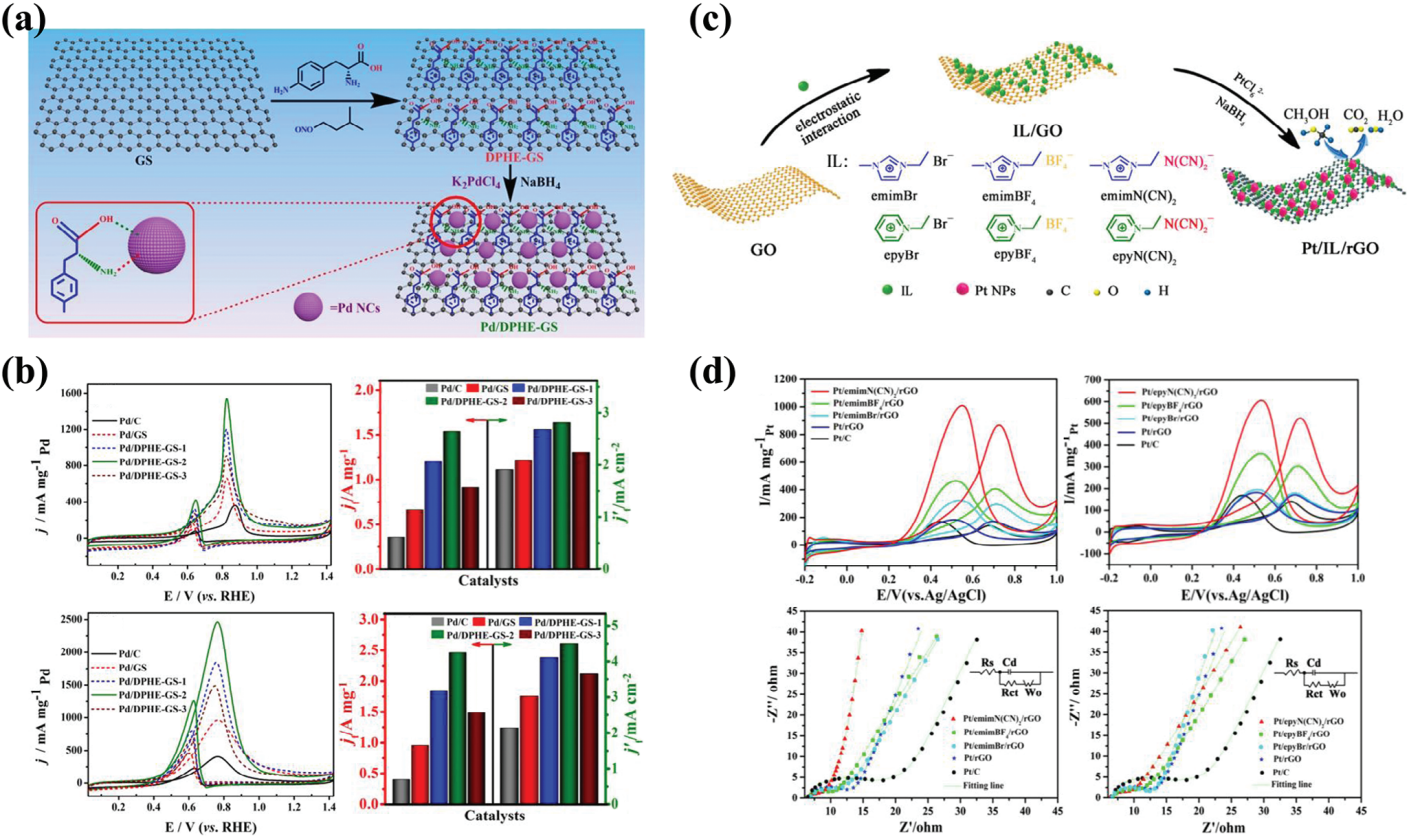
a) Schematic diagram of the fabrication of the Pd/DPHE‐GS. b) CV curves, and j_f_ (mass activity), and j_f_’(specific activity) histograms of the tested‐electrocatalysts. Reproduced with permission.^[^ ^89^ ^]^ Copyright 2021, Elsevier. c) Schematic Illustration of the Synthesis Process of the Pt/IL/rGO Catalysts. d) CV curves and Nyquist plots of catalysts. Reproduced with permission.^[^ ^100^ ^]^ Copyright 2021, American Chemical Society.

The introduction of organic molecules containing functional groups into graphene can serve as an effective stabilizer to reduce the size and improve the uniformity of the grown metal NPs.^[^ ^88^ ^]^ Furthermore, the metal‐organic interface interactions resulted from the strong interactions between the functional groups of the organic molecules and the metal NP can effectively modulate the surface structure and electron density of the metal NPs.^[^ ^98^ ^]^ Shuwen Li's group performed many impressive studies in this respect. For instance, they chose D‐phenylalanine as a functional organic molecule to synthesize functionalized graphene by diazotization reaction under mild conditions, and the abundant amino and carboxyl groups on the organic molecule can provide attachment sites for Pd NPs (**Figure** **6**a).^[^ ^89^ ^]^ It is commendable that the related catalytic performance of the catalysts in alkaline environments for MOR and EOR was indeed significantly improved. For example, the Pd‐based catalyst shows the highest electrocatalytic activity (1539.0 mA mg^−1^, 2.81 mA cm^−2^, and 2464.8 mA mg^−1^, 4.50 mA cm^−2^, respectively) and the most excellent long‐term durability and stability toward MOR and EOR compared to the commercial Pd/C and other counterparts (Figure 6b). In addition, other functional organic molecules containing amino and carboxyl groups, such as D‐4‐amino‐phenylalanine,^[^ ^88^ ^]^ aniline,^[^ ^42^ ^]^ and 4‐aminothiophenol,^[^ ^99^ ^]^ have been extensively studied, and great progress has been made.

The non‐covalent functionalization of graphene has also received particular attention because it allows for molecular attachment through supramolecular interactions (such as *π*–*π* stacking, electrostatic interactions, and hydrogen bonding) and thus maintains the unique structure and the excellent performance of graphene.^[^ ^87^ ^]^ Non‐covalent functionalized GNTs were successfully synthesized via solvent‐exfoliation of expanded graphite with the aid of supercritical CO_2_ and 1‐pyrenylamine (PA).^[^ ^101^ ^]^ Furthermore, the material was exploited as a support for metal Pt to prepare PA‐GNS/Pt catalyst for MORs. In the obtained catalyst, Pt NPs supported on PA‐GNS exhibited higher ECSA (82.9 m^2^ g^−1^), better methanol catalytic activity (365.6 mA mg^−1^), and long‐term stability (retains 75.0% of the initial ECSA after 500 cycles) compared with the commercial catalysts (JM‐C/Pt and GNS/Pt). Similar approaches were also reported by Fan et al. and Ma et al., employing porphyrin and viologen derivatives as noncovalent functional organic molecules, respectively.^[^ ^102^, ^103^ ^]^

Another approach is the combination of ILs and graphene (Figure 6c).^[^ ^100^ ^]^ The noncovalent functionalization of graphene with IL enhances its electrical conductivity and accelerates the charge transfer pathway, thereby greatly improving the electrocatalytic performance and stability of the catalysts for AORs. Moreover, the presence of ILs could stabilize the metal NPs for high catalytic properties. Compared with Pt/C and Pt/rGO catalysts, the ILs‐functionalized Pt/IL/rGO catalysts have more active sites and lower charge transfer resistance, resulting in better electrocatalytic activity (Figure 6d). Furthermore, it can also shift the d‐band center of Pd down through strong electronic interactions, which enhances the durability of CO‐like intermediates and electrocatalytic reaction kinetics for EOR.^[^ ^40^ ^]^

#### 4.1.2 Heteroatom‐Doping

It is very important to enhance the catalytic performance for AORs of supported metal catalysts by heteroatom‐doping to adjust the electronic structure of the 2D materials. For heteroatom‐doped carbon materials, heteroatoms are believed to induce charge density and/or spin density redistribution on the adjacent carbon atoms, which alters the electronic structure and d‐band center of the active metal due to strong electronic interaction between supports and metals.^[^ ^104^, ^105^ ^]^ This alteration affects the free energy of adsorption/desorption of carbonaceous intermediates by the metal during the catalytic reaction and enhances the AOR catalytic activity. There are two mainly types of heteroatom‐doped graphene support: single‐element doping and multi‐element co‐doping. Heteroatoms can be incorporated into graphenes directly during the synthesis process (in situ doping) or by post‐treatment (post‐doping). In situ doping refers to the direct pyrolysis of precursors containing carbon and heteroatoms at high temperatures to achieve heteroatoms uniformly incorporated into the carbon matrix. The incorporation of heteroatoms into existing support by introducing a heteroatom precursor and post‐processing is known as a post‐doping method. In situ doping typically has higher doping content compared to post‐doping. Heteroatoms commonly used to dope graphene include one or more of nitrogen, boron, phosphorus, sulfur, etc.

**Figure 7:**
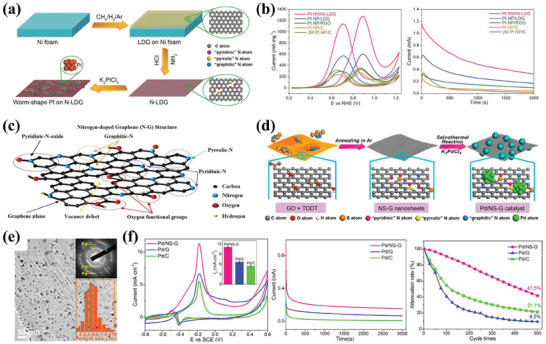
a) Illustration of the synthesis of Pt NW/N‐LDG catalyst. b) Cyclic voltammograms and Current‐time curves of the different electrodes. Reproduced with permission.^[^ ^94^ ^]^ Copyright 2017, Wiley‐VCH. c) N‐doped graphene nanomaterial structure. Reproduced with permission.^[^ ^106^ ^]^ Copyright 2021, Elsevier. d) Schematic of the Synthetic Procedures for the Pd/NS‐G catalyst. e) TEM images of the Pd/NS‐G catalyst. f) CVs, Chronoamperometric curves, and Cycling stability of the Pd/NS‐G, Pd/C and Pd/G electrodes. Reproduced with permission.^[^ ^95^ ^]^ Copyright 2016, American Chemical Society.

Among the reported heteroatom dopants, nitrogen (N) is considered a very attractive element for doped materials.^[^ ^106^, ^107^, ^108^ ^]^ In the periodic table, N is a VA group element with a close atomic radius to C atoms, which could easily substitute C atoms in graphene to form N‐doped graphene materials. Since the electronegativity of N atoms is stronger than C atoms, they can get electrons from neighboring C atoms, thus inducing the formation of positively charged C atoms. These positively charged C atoms could modulate the electronic structure and d‐band centers of the loaded metal NPs, which resulted in excellent electrochemical and catalytic performance for AORs of the N‐doped graphene‐supported metal catalysts.^[^ ^109^, ^110^, ^111^, ^112^ ^]^ Also, the interaction between metal NPs and graphene support was greatly strengthened compared to pristine graphene, which ultimately contributed to the substantial improvement of catalytic activity and stability.^[^ ^92^, ^113^ ^]^ Compared with the graphene‐supported Pt catalysts, the prepared N‐doped graphene‐supported Pt catalysts exhibited higher mass activity (1283.1 mA mg^−1^) and ECSA (60.9 m^2^ g^−1^) toward MORs with reliable long‐term stability and excellent antipoisoning ability by the bifunctional mechanism (**Figure** **7**a,b).^[^ ^94^ ^]^

The strategy of using N‐doped graphene to improve the catalyst performance has been widely applied in AOR research.^[^ ^114^, ^115^, ^116^ ^]^ It was found that different bonding configuration of doped nitrogen atoms with graphene change the structure and electronic configurations of the material in significantly different ways.^[^ ^106^ ^]^ Common bonding configurations of doped nitrogen atoms include pyrrolic‐N, pyridinic‐N, graphitic‐N, and pyridinic‐N oxide (Figure 7c). It was found that pyridinic‐N bonding configurations could better facilitate the growth of Pt on graphene supports, which enhances the catalytic activity of Pt NPs toward the MOR.^[^ ^117^ ^]^ And graphitic‐N and pyridinic‐N are the key sites for ORR catalysis on N‐doped graphene nanomaterials.^[^ ^57^ ^]^ In MORs, the pyrrolic‐N and pyridinic‐N were suggested to exhibit the ability to promote the adsorption and oxidation of CH_3_OH and the oxidation of CO_ads_.^[^ ^118^ ^]^

Boron (B), sulfur (S), and phosphorus (P) doping was also explored to tune the electronic structure of graphene.^[^ ^119^ ^]^ In comparison with nitrogen, boron (2.04) and phosphorus (2.19) have lower electronegative than carbon and Pt and thus donate electrons that induce a negative shift in the d‐band center of metal and a weakening of CO adsorption. Du et al. prepared B‐doped graphene‐supported Pt (Pt/BG) catalysts by thermally annealing the mixture of GO and boric acid.^[^ ^120^ ^]^ The obtained catalysts have lower d‐band centers due to B doping, which weakens the absorption of the CO poisoning intermediates which contributes to the long‐term stability and activity of the Pt/BG catalysts. The corresponding P‐doped graphene supports were explored, namely, the prepared Pt/PG (P‐doped graphene) catalyst exhibited over 1.54 times enhanced methanol electrooxidation activity compared with Pt/G, as well as desirable stability and remarkably improved CO‐tolerance.^[^ ^121^ ^]^ However, it is difficult to introduce P into the graphene because of its large atomic radius. Recent reports suggest that introducing metalloid phosphorous into Pt‐M alloy to form a Pt‐M‐P structure is a promising method for P doping.^[^ ^93^ ^]^ Unlike other dopants, sulfur (2.58) and carbon (2.55) have similar electronegativity, and the covalent bond between S and C atoms is essentially non‐polarized with negligible electron transfer. A plausible explanation for the enhanced electrocatalytic activity is that sulfur dopant modifies the electronic structure of graphene by inducing a non‐uniform spin density distribution.^[^ ^122^ ^]^ Furthermore, it can effectively facilitate the GO reduction and increase the electrical conductivity of rGO.^[^ ^123^ ^]^ The S‐doped graphene contains two types of S‐bonding configurations (thiophene‐S and oxidized S). Due to the different bond lengths of the C─S and C─C bonds, they are more inclined to form thiophene sulfur, leading to the improved AOR performance of the catalysts with the S‐doped graphene support.^[^ ^124^ ^]^

Co‐doping of graphene with multiple elements could provide much more electroactive sites due to the efficient synergistic effect, thus further enhancing the catalytic performance.^[^ ^125^, ^126^ ^]^ For instance, the Pd NPs were supported on N and S dual‐doped graphene as a catalyst for AORs.^[^ ^95^ ^]^ The NS‐G sheets could provide sufficient anchoring sites to grow highly uniform Pd NPs (Figure 7d). These well‐designed Pd/NS‐G catalysts have numerous Pd electroactive sites with high catalytic activity and abundant hydroxyl species (produced by N and S atoms) for the oxidation of CO‐like poisoning intermediates. The Pd/NS‐G had a high ECSA value (103.6 m^2^ g^−1^), outstanding catalytic ability (11.3 mA cm^−2^ and 399.3 mA mg^−1^), and excellent long‐term electrocatalytic stability (Figure 7e). Similarly, the N and P, N and B dual‐doped graphene support were reported by our group.^[^ ^127^, ^128^ ^]^ Namely, the synergistic doping of N and P changed the electronic structure of graphene, increased the electron density of Pd, strengthened the bonding of Pd NPs with graphene, and improved the catalytic activity and stability for methanol oxidation. In addition, co‐doping with different elements has been extensively studied, leading to significant progress.^[^ ^129^, ^130^, ^131^ ^]^

#### 4.1.3 Composite Hybridization

**Figure 8:**
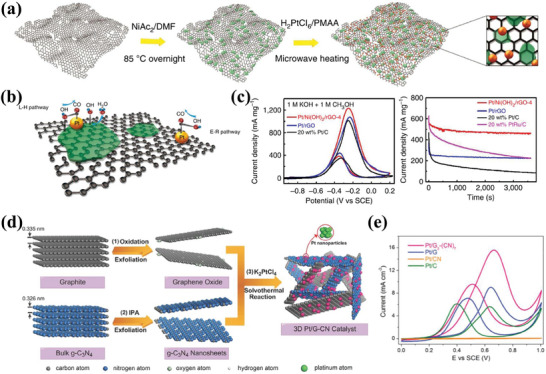
a) A schematic illustration of the two‐step solution method to prepare the ternary hybrid materials. b) A schematic illustration showing the bifunctional interaction between Ni(OH)_2_ and adjacent Pt sites for the dissociate adsorption of water molecules and subsequently the oxidative removal of CO on Pt sites via the L‐H pathway. c) CV curves and Short‐term durability measurement. Reproduced with permission.^[^ ^133^ ^]^ Copyright 2015, Springer Nature. d) Illustration of the synthesis of the 3D Pt/G‐CN catalyst. e) CV curves of the Pt/G‐CN architectures. Reproduced with permission.^[^ ^49^ ^]^ Copyright 2014, Wiley‐VCH.

Composite materials enable unexpected complementary advantages and sometimes achieve the effect that “1 + 1 > 2”, similar to the high catalytic activity and stability of PtRu alloys.^[^ ^132^ ^]^ As a simple and classic example, Li et al. reported a Pt‐based hybrid support based on graphene and Ni hydroxide (**Figure** **8**a).^[^ ^133^ ^]^ Among them, the OH_ads_ species provided by Ni hydroxide can facilitate the oxidative removal of carbonaceous poisonous on adjacent active metal sites of catalyst, and the graphene could compensate for the drawbacks of transition‐metal hydroxides by providing the high electrical conductivity required for fast electrocatalysis (Figure 8b). Based on the reasonable design, the prepared hybrid support catalyst Pt/Ni(OH)_2_/rGO exhibited both remarkable activity and durability for MORs, and its current density was maintained at 4460 mA mg^−1^ after 1 h of operation (Figure 8c). Moreover, The authors discovered that Pt/Ni(OH)_2_/rGO could be reactivated by cyclic voltammetry (CV) to recover the full MOR activity to its initial value after durability measurement. Similarly, this strategy was also used to improve the catalytic performance of EOR, and it was revealed that the incorporation of Ni(OH)_2_ significantly changed the selectivity of ethanol oxidation from the originally predominant C2 pathway to the more desirable C1 pathway.^[^ ^96^ ^]^ And hybridizing graphene with metal oxides could also be achieved for this purpose, for example, such as the widely reported graphene hybridized supports based on SnO_2_, TiO_2_, and CeO_2_.^[^ ^134^, ^135^, ^136^, ^137^, ^138^ ^]^ Another approach that is worthy of attention involves combining graphene and black phosphorus (BPs).^[^ ^139^ ^]^ It has been shown that this heterostructure not only has a strong electronic interaction with Pd making it easy to absorb OH radicals for removing CO_ads_ intermediate to release active sites on EOR, but also serves as an excellent site for H_2_O dissociative adsorption, which facilitates the generation of addition OH_ads_ for the oxidation of carbonaceous poison intermediates. This strategy of using composites to enhance the catalytic performance of support metal catalysts has been widely used in AOR research.

It is well‐known that during the reaction process, individual graphene sheets could irreversibly agglomerate due to the intensive van der Waals forces, which reduce the surface area and blocks the active sites.^[^ ^140^ ^]^ This problem could be effectively relieved by compositing graphene with other materials or preparing graphene aerogels to construct 3D structures.^[^ ^141^, ^142^ ^]^ The main materials being used as composites include CNTs, g‐C_3_N_4_, MXene, and conductive polymers.^[^ ^143^, ^144^, ^145^ ^]^ For example, 3D architectures based on graphene and g‐C_3_N_4_ nanosheets were used to support Pt as a catalyst for methanol oxidation (Figure 8d).^[^ ^49^ ^]^ The 3D architectures not only provide large, accessible multi‐sized pores and high electrical conductivity but are also conducive to preventing the agglomeration of GNTs. Thus, the 3D Pt/G‐CN shows superior electrocatalytic properties toward methanol oxidization reaction (Figure 8e). To achieve this goal, graphene and MXene (Ti_3_C_2_T_x_) nanosheets were combined as support materials.^[^ ^47^ ^]^ Additionally, the aggregation problem of graphene support during the catalytic reaction by preparing graphene aerogels can also be effectively alleviated, thus improving the overall catalyst durability.^[^ ^146^ ^]^ The studies show that graphene aerogels can not only accelerate mass transfer but also provide a larger efficient surface area for methanol oxidation.^[^ ^130^, ^147^ ^]^ These researches are of great significance to the development of graphene‐based support materials.

### 4.2 MXenes

**Figure 9:**
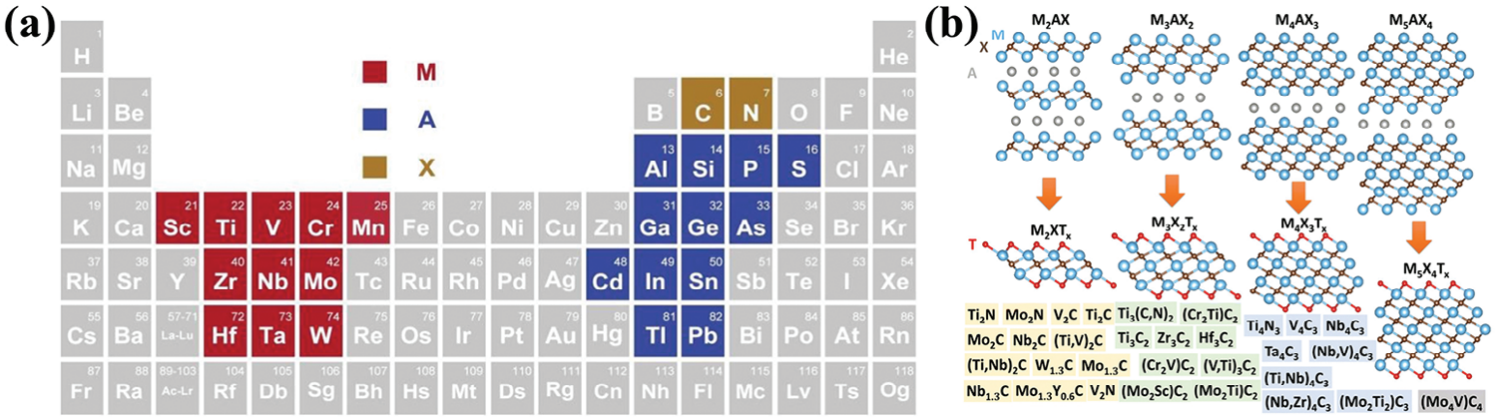
a) The position of MAX phase in the periodic table of elements. Reproduced with permission.^[^ ^161^ ^]^ Copyright 2019, Elsevier. b) Schematic illustration of the structures of corresponding MAX phases. Reproduced with permission.^[^ ^162^ ^]^ Copyright 2023, The Royal Society of Chemistry.

As a new member of the 2D family, MXenes are layered structural materials composed of transition metal carbides, nitrides, and carbonitrides.^[^ ^148^, ^149^ ^]^ Since Barsoum et al. discovered Ti_3_C_2_ as the first MXene in 2011,^[^ ^150^ ^]^ a variety of MXenes have been invented and extensively explored for energy conversion and storage.^[^ ^151^, ^152^, ^153^, ^154^ ^]^ It has also been explored as possible non‐carbon support for depositing metal NPs for AORs due to its unprecedented physicochemical characteristics.^[^ ^155^, ^156^ ^]^ MXene is acquired by the selective removal of the “A” layer from the MAX phase of M_n+1_AXn with general notation (M_n+1_X_n_T_x_), where “M” denotes transition metals; “A” refers to an element from Al, Si, Cd, P, Ga, Ge, As, In, Sn, Pb, or S (in general, Al or Si); “X” stands for carbon and/or nitrogen, and “T” stands for the surface termination groups (‐O, ‐OH, and ‐F) (**Figure** **9**).^[^ ^157^ ^]^ After a decade of development, their current synthesis methods can be categorized into five types: thermal treatment, hydrothermal/solvothermal, low‐temperature soft‐chemical, electrochemical, and physical.^[^ ^155^, ^158^ ^]^ The synthesis method of MXene has a significant effect on its surface and electronic structure, especially the surface termination groups. Further, the terminated surfaces of MXene will affect the performance of the electrocatalytic reaction.^[^ ^159^, ^160^ ^]^ Owing to their large surface area, high conductivity, excellent electrochemical and thermal stability, and good hydrophilicity properties, these structured 2D MXenes with tunable surface chemistry open new avenues for the designing of highly catalytic activity and long‐life supported metal catalyst materials for AORs.

The high surface area and suitable layer spacing of MXene are beneficial for the dispersion of Pt NPs with ultrafine sizes; at the same time, the strong electronic interactions between the metal and the support could modulate the electronic structure of the loaded metal and fully play to their synergistic coupling effect.^[^ ^163^, ^164^, ^165^ ^]^ Unlike typical carbon support, MXene exhibits good thermal stability and maintains good performance at both elevated temperatures and potential.^[^ ^166^ ^]^ Metals with electrocatalytic activity for AOR, such as Pt, Pd, and Ni, can be used to prepare MXene‐based metal catalysts, thus improving their catalytic performance for AOR.^[^ ^167^, ^168^ ^]^ For instance, Zhou et al. reported metal Pd catalysts (Pd/MXene) with 2D Ti_3_C_2_T_x_ MXene as the support for MOR.^[^ ^169^ ^]^ The prepared Pd/MXene exhibits a high MOR current density (12.4 mA cm^−2^), superior to the commercial Pd/C (7.6 mA cm^−2^). Density functional theory (DFT) calculations revealed that there was a SMSI effect between MXene support and Pd NPs, where electrons transfer from MXene support to Pd NPs. This process had a favorable influence on the surface electronic structure of the Pd NPs, which optimized their adsorption performance on methanol and, therefore, contributed to the improvement of MOR activity. In addition, the introduction of the MXene support can promote the formation of hydrogen bonds between the methanol molecules and the support terminations, thus improving the oxidation kinetics of MOR. Similarly, Huang et al. fabricated Ti_3_C_2_T_x_ MXene‐loaded Pt nanowire catalysts (Pt NWs/MXene).^[^ ^38^ ^]^ The adsorption energy of Pt NWs on the MXene support was 1.55 eV per atom higher than that of Pt NWs on a carbon support, which proved the strong interaction between the Pt NWs and MXene. Meanwhile, the d‐band center of Pt NWs grown on MXene (−2.45 eV) downshifted by ≈0.34 eV compared with that of Pt NWs grown on carbon substrate (−2.11 eV). Compared with Pt NWs/C, Pt NWs/rGO catalysts, Pt NWs/MX shows superior methanol oxidation performance, including high ECSA value and MOR activity, outstanding toxicity resistance, and excellent durability.

**Figure 10:**
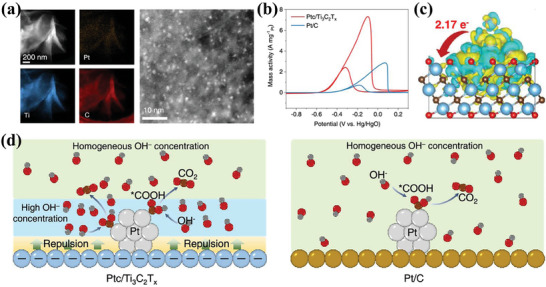
a) HAADF‐STEM image and spectrometry mapping of Ptc/Ti_3_C_2_T_x_. b) Mass activity of Ptc/Ti_3_C_2_T_x_ and Pt/C. c) Charge density difference, where the yellow and blue areas denote the electron accumulation and depletion on Ptc/Ti_3_C_2_T_x_. d) Schematic illustration of the proposed MOR mechanism on Ptc/Ti_3_C_2_T_x_ and Pt/C. Reproduced with permission.^[^ ^170^ ^]^ Copyright 2022, American Chemical Society.

Besides exploiting the structural advantages to modulate the microstructure and interactions of metal NPs, the use of MXene as support also benefited the improvement of the tolerance of catalyst to CO poisoning, which thus exhibits ultrahigh MOR activity and durability.^[^ ^171^ ^]^ Wang, Mai, and coauthor prepared Ti_3_C_2_T_x_ MXene modified with ultrafine Pt clusters (Ptc/Ti_3_C_2_T_x_) by spray‐drying method, which exhibited the highest mass‐specific activity (7.32 A mg_Pt_^−1^) for MOR (**Figure** **10**a,b).^[^ ^170^ ^]^ The charge density difference revealed that 2.17 electrons were transferred from the Pt clusters to the Ti_3_C_2_T_x_ substrate, and therefore, the Pt clusters were positively charged while the Ti_3_C_2_T_x_ substrate was negatively charged (Figure 10c). The authors suggest that the electron‐rich Ti_3_C_2_T_x_ substrate generates a strong surface electric field, which will promote the hydroxyls away from the substrate, leading to a high hydroxyl concentration around the Pt clusters, promoting further oxidation of CO_ads_, and thus achieving higher MOR activity and durability (Figure 10d). This research is of great significance to the development of MXene‐based metal catalysts. In addition to Ti_3_C_2_T_x_ MXene, 2D Fe‐based MXene (Fe_3_C_2_) nanosheets have also been reported to be used as metal catalyst support for AORs.^[^ ^172^ ^]^

Additionally, MXene can be designed and regulated through various strategies such as surface termination, heteroatom doping, and complex hybridization to modulate the catalytic centers of the catalysts further, thus promoting their application in alcohol electrocatalytic energy conversion systems. The recent advances in MXene‐supported metal catalysts for AOR have been summarized in **Table** **3**.

**Table 3:** Summary of the MXene‐based metal catalysts for the AOR.

Catalyst	Electrolyte	ECSA [m^2^ g^−1^]	Mass activity [mA mg^−1^]	Specific activity [mA cm^−2^]	Stability (Retention rate)	Refs.
Pt/Ti_3_C_2_T_x_	1.0 m CH_3_OH + 0.5 m H_2_SO_4_	105.5	1621.5	1.6	71.1% after 500 cycles	[38]
Rh/Ti_3_C_2_T_x_	1.0 m CH_3_OH + 1.0 m KOH	71.6	600.2	0.84	‐	[176]
Pt‐Ni/Ti_3_C_2_	1.0 m CH_3_OH + 0.1 m HClO_4_	61	1377.5	2.25	84.7% after 400 cycles	[177]
Pt‐Pd/Ti_3_C_2_T_x_	1.0 m CH_3_OH + 0.5 m H_2_SO_4_	157.3	1461.7	0.93	62.9% after 200 cycles	[171]
PtRhFe/Ti_3_C_2_T_x_	1.0 m C_2_H_5_OH + 1.0 m KOH	46.4	3407.7	7.3	72.8% after 1000 cycles	[178]
PdCu/N‐Ti_3_C_2_T_x_	1.0 m CH_3_OH + 1.0 m KOH	36.4	2200.7	13.1	43.2% after 500 cycles	[179]
PdSn_0.5_/Se‐Ti_3_C_2_	1 m CH_3_OH + 1.0 m NaOH	76.2	4762.8	‐	‐	[180]
Pd/B‐N‐Ti_3_C_2_	1.0 m C_2_H_5_OH + 1.0 m KOH	83.1	937.2	1.12	81.4% after 400 cycles	[45]
Pd/DMAB‐Ti_3_C_2_	1.0 m C_2_H_5_OH + 1.0 m KOH	15.2	535	‐	74.6% after 350 cycles	[41]
Pt/PDDA‐Ti_3_C_2_T_x_	1 m CH_3_OH + 0.5 m H_2_SO_4_	61.0	607.6	‐	‐	[181]
Pd/PDDA‐Ti_3_C_2_T_x_	1 m CH_3_OH + 0.5 m NaOH	105.3	1526.5	1.5	‐	[39]
Pt/RGO‐Ti_3_C_2_T_x_	0.5 m CH_3_OH + 0.5 m H_2_SO_4_	90.1	1102.0	1.2	24.4% after 1000 cycles	[47]
Pt/Ti_3_C_2_T_x_‐MWCNTs	2.0 m CH_3_OH + 1.0 m H_2_SO_4_	175.0	922.0	0.15	13.3% after 100 cycles	[182]
Pd/Ti_3_C_2_T_x_‐rGO	1.0 m CH_3_OH + 1.0 m KOH	97.97	753	25.47	67% after 300 cycles	[183]
Pd/GO‐Ti_3_C_2_T_x_‐PS	1.0 m C_2_H_5_OH + 1.0 m KOH	89.9	2944.0	‐	55.4% after 800 cycles	[184]
Pd/Ti_3_C_2_T_x_‐NG	1.0 m C_2_H_5_OH + 1.0 m KOH	34.5	2262.2	6.56	31.4% after 500 cycles	[185]
Pt‐CoP/CNT‐Ti_3_C_2_T_x_	1.0 m CH_3_OH + 0.5 m H_2_SO_4_	97.70	1704	1.76	50.8% after 1000 cycles	[186]
Rh/ZIF‐Ti_3_C_2_T_x_	1.0 m CH_3_OH + 1.0 m KOH	161.5	2955.1	1.83	68.2% after 500 cycles	[187]
Pd/ZIF‐Ti_3_C_2_T_x_	1.0 m C_2_H_5_OH + 1.0 m KOH	96.2	2237	2.23	‐	[188]

#### 4.2.1 Surface Terminations and Functionalization

Unlike graphene exfoliation, the MAX cannot be exfoliated easily due to the strong interaction between the transition metal and “A”, such as Al and Si, in the compound.^[^ ^173^ ^]^ MXenes are currently mainly prepared by selective etching of the “A” layer in MAX phases by aqueous solutions containing fluoride ions, such as HF or the mixtures of LiF and HCl. During the production or etching process, the surface of MXenes is inevitably exposed to the solvent environment, enabling it to be decorated with “T” terminations. The most common species of these “T” terminations are ‐O, ‐OH, and ‐F. However, it is worth noting that negatively charged fluorine‐containing functional groups are not beneficial for the adsorption of metal precursors (such as PtCl_4_^2−^, PdCl_4_^2−^, PdCl_6_^4−^, etc.) due to electrostatic repulsion. The different preparation strategies for MXenes may introduce different terminations, thus affecting their properties and performance.^[^ ^174^, ^175^ ^]^ Therefore, the rational design of the type, content, and distribution of surface terminations is crucial to improve the catalytic performance of AOR.

In addition to the alteration of terminal groups, another approach that is worthy of attention is to tune the surface charge property of MXene nanosheets by surface modification based on scalable noncovalent strategy^[^ ^189^ ^]^ and thus achieve a homogeneous distribution of metal NPs, as reported by Weihua Li, Huajie Huang, and co‐workers.^[^ ^181^ ^]^ In this paper, the authors chose poly(diallyl dimethyl‐ammonium chloride) (PDDA) as the water‐soluble cationic to adjust the surface charge properties of Ti_3_C_2_T_x_ nanosheets. The positively charged polyelectrolyte long chains PDDA enhanced the electrostatic interactions between Ti_3_C_2_T_x_ nanosheets and metal precursors, inducing the formation of 1D worm‐shaped Pt nanocrystals and also preventing the aggregation or detachment of Pt from the support during the reaction process. The prepared Pt NWs/PDDA‐Ti_3_C_2_T_x_ catalysts exhibit superior electrocatalytic ability over Pt NPs loaded on MXene substrates, including high specific activity, extraordinary antipoisoning capacity, and good electrocatalytic stability for MOR. Furthermore, the attachment of redox‐active groups such as 4‐amino‐TEMPO (2,2,6,6‐tetramethylpiperidine‐1‐oxyl) to MXene based on covalent bonding strategies can also have a significant influence on its electrochemical performance.^[^ ^190^ ^]^

#### 4.2.2 Heteroatom Doping

Based on the characteristics of the dopant atomic structure, elemental doping can modulate the electronic properties of the material itself by performing surface modification or lattice substitution on the pristine MXene.^[^ ^191^, ^192^ ^]^ In recent years, optimization of pristine MXene properties (such as electronic, optical, magnetic, etc.) by means of elemental doping has been demonstrated to be an effective strategy.^[^ ^193^, ^194^ ^]^ The incorporation of heteroatoms adjusts the coordination environment of the MXene surface, which in turn affects the adsorption strength toward reaction intermediates. For AORs, the element‐doped MXene material as support offers the following advantages: 1) providing more active metal anchor sites and influencing the metal microstructure; 2) interacting strongly with the metal to modulate its d‐band center; 3) immobilizing the active metal to prevent migration or detachment during the reaction process; 4) improving the conductivity of the material. To this end, elemental doping is one of the most effective methods to improve the structure of MXene carriers. In MXene support, common doping elements include N, B, Se, etc.

**Figure 11:**
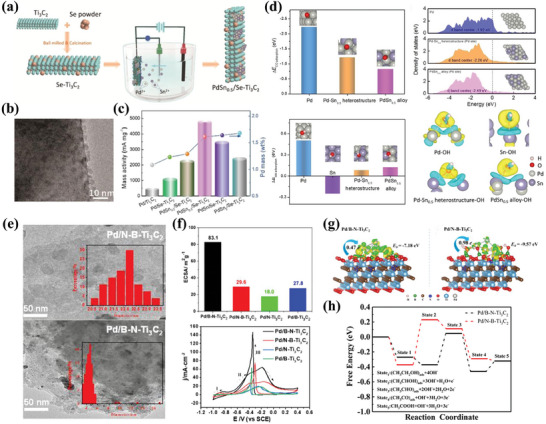
a) Schematic illustration of deposition process of Se‐Ti_3_C_2_ immobilized PdSn_0.5_ nano‐alloy. b) TEM images of PdSn_0.5_/Se‐Ti_3_C_2_. c) The maximum mass activity of the corresponding MORs. d) DFT calculations. Reproduced with permission.^[^ ^180^ ^]^ Copyright 2022, Wiley‐VCH. e) TEM image of Pd/N‐B‐Ti_3_C_2_ and Pd/B‐N‐Ti_3_C_2_. f) The specific ECSA values and CV curves of catalysts at 1 m CH_3_CH_2_OH/KOH at 50 mV s^−1^. g) The electron transfer quantity of the Pd and Ti_3_C_2_ support. h) DFT calculated free energy profiles of EOR. Reproduced with permission.^[^ ^45^ ^]^ Copyright 2022, American Chemical Society.

Engineering MXenes with single‐element heteroatoms is an effective strategy to enhance the electrocatalytic activity of support metal catalysts. Zeng et al. prepared palladium‐tin (PdSn) nano‐alloy catalysts (PdSn_0.5_/Se‐Ti_3_C_2_) with Se‐doped MXene as the support via a progressive one‐step electrochemical deposition strategy and investigated their catalytic performance for methanol oxidation in detail (**Figure** **11**a–c).^[^ ^180^ ^]^ The use of Se‐doped Ti_3_C_2_ MXene as support improves the deposition of active metals. Experimental and theoretical calculations show that the strong electronic interactions between metal and Se‐doped MXene, as well as the optimized distribution of Pd‐Sn active sites, can modulate the d‐band centers, decrease the adsorption energy of CO_ads_ on the Pd sites, and enhance the generation of OH_ads_ on the Sn sites (Figure 11d). This well‐designed PdSn_0.5_/Se‐Ti_3_C_2_ catalyst exhibits higher MOR mass activity (4762.8 mA mg^−1^) and excellent long‐term stability (Figure 11c). Our group has performed some research on single‐element‐doped MXene‐based metal catalysts. In 2021, N‐doped Ti_3_C_2_ support Pd‐based catalysts (Pd/N‐Ti_3_C_2_) were prepared with NH_3_·H_2_O as the N source.^[^ ^195^ ^]^ DFT calculation showed that the number of electron transfer and electron binding energy between the N‐doped Ti_3_C_2_ carrier and Pd are higher than those between undoped Ti_3_C_2_ and Pd. The Pd/N‐Ti_3_C_2_ catalyst shows higher ECSA, ethanol oxidized current densities, and electrochemical stability than the Pd/ Ti_3_C_2_. MXene, based on the doping of other elements, has been widely used in catalyzes such as hydrogen evolution reaction (HER), oxygen evolution reaction (OER), and ORR.^[^ ^196^ ^]^ However, it has not been reported in MORs. Under the background of the current lack of electrocatalysts for AORs, the development of more element‐doped MXene‐supported metal catalysts to meet the demand is particularly necessary.

Co‐doping in MXenes is also an effective method to adjust the AOR catalytic activity of supported metal catalysts. For instance, B and N co‐doped Pd‐loaded Ti_3_C_2_ (Pd/DMAB‐Ti_3_C_2_) catalysts were prepared by a rapid, one‐step hydrothermal method using dimethylamine borane (DMAB) as both heteroatom dopant and metal‐reducing agent.^[^ ^41^ ^]^ The experimental results showed that the co‐doped catalyst Pd/DMAB‐ Ti_3_C_2_ showed much higher ECSA, ethanol oxidation current density, and electrochemical stability than the single‐doped catalyst. This excellent property can be attributed to the synergistic effect of the two dopant atoms, B and N, which significantly affects the electron transfer and d‐energy band centers of the loaded metal Pd, thereby weakening the adsorption of CO derivatives and thus enhancing the EOR performance. Meanwhile, the element doping sequence in co‐doping also affects the electronic structure of MXene support, which influences the electrocatalytic performance of the supported‐metal catalysts for AORs. Two MXene‐based metal catalysts, Pd/B‐N‐Ti_3_C_2_ and Pd/N‐B‐Ti_3_C_2_, were synthesized with different doping sequences to evaluate the effect of the B and N co‐doping sequences on the electronic structure of the metal surface as well as the electrocatalytic performance of the EOR.^[^ ^45^ ^]^ The metal Pd NPs of Pd/B‐N‐Ti_3_C_2_ is better distributed on the MXene support with a much smaller diameter (≈4.4 nm) than that of Pd/N‐B‐Ti_3_C_2_ (≈22 nm) (Figure 11e). Meanwhile, the electronic binding energies of both substrate and Pd in Pd/B‐N‐Ti_3_C_2_ are smaller than that of Pd/N‐B‐Ti_3_C_2_, and the B and N doping order sequence affects the electronic structure of the supported metal Pd (Figure 11f). Therefore, Pd/B‐N‐Ti_3_C_2_ has higher ECSA, electrocatalytic activity, and antitoxicity of CO_ads_ than Pd/N‐B‐Ti_3_C_2_. Moreover, DFT calculations showed that different B‐doped species could provide sites for H atoms from CH_3_CH_2_OH of dehydrogenation in Pd/B‐N‐Ti_3_C_2_, thus improving the catalytic activity for EOR (Figure 11g,h).

#### 4.2.3 Composite Hybridization

Similar to other 2D supports, MXene‐based metal catalysts tend to recolonization or restack during the reaction process due to their high surface energy, leading to a drastic loss of catalytically active sites.^[^ ^197^ ^]^ Furthermore, its poor charge transfer performance is also unfavorable to the catalytic reaction. To alleviate these problems, the preparation of 3D MXene or the introduction of “spacers” (for example, graphenes, metal‐organic frameworks (MOFs), conductive polymers, etc.) into the MXene sheets is considered an efficient approach.^[^ ^198^ ^]^ These 3D hybrids could effectively prevent the aggregation of MXene, ensuring high surface area, high conductivity, and rich metal anchor sites.

The strategy of directly preparing 3D MXene support to improve the overall performance of catalysts has wide attention in the field of electrocatalytic energy. For instance, Wang et al. prepared MXene Ti_3_C_2_T_x_ with 3D crumpled balls as support for loaded Pt NPs by a spray‐drying process.^[^ ^170^ ^]^ The 3D structure effectively inhibits the agglomeration of MXene Ti_3_C_2_T_x_, maximizes the exposure of Pt sites, and enlarges the contact area between the catalyst and electrolyte, resulting in ultrahigh MOR activity and durability. Another classical method for preparing 3D MXene frameworks is the template method.^[^ ^199^ ^]^ 3D interconnected porous structures of MXene were constructed using the sacrificial template of polystyrene microspheres and then explored as a support to prepare Pt‐based catalysts.^[^ ^200^ ^]^ The prepared 3D Pt/e‐MXene catalysts effectively prevented the aggregation of MXene during the reaction process and achieved rapid electro/ionic transport, thus resulting in a significant improvement in electrocatalytic activity and long‐term stability during ethanol oxidation.

**Figure 12:**
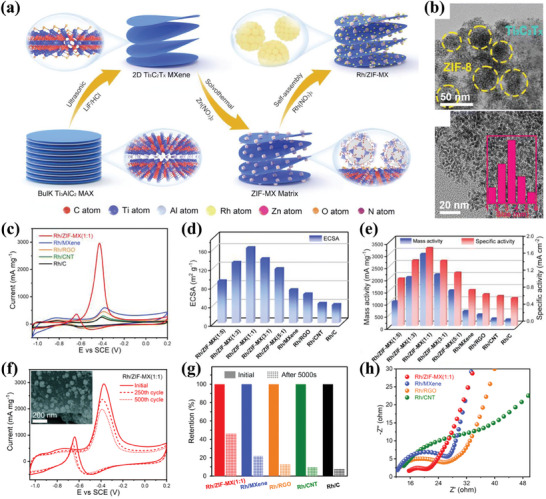
a) The schematic of the synthetic route of the Rh/ZIF‐MX nanoarchitecture. b) The TEM of the Rh/ZIF‐MX nanoarchitectures. c) The CV curves of diverse electrocatalysts. d) The specific ECSA values of the diverse electrocatalysts. e) The mass and specific activities of the diverse electrocatalysts. f) The CV curves of Rh/ZIF‐MX before and after 500 cycles. g) The methanol oxidation mass activities of the diverse electrodes before and after the chronoamperometry test. h) The AC impedance spectra of diverse electrocatalysts. Reproduced with permission.^[^ ^187^ ^]^ Copyright 2023, Royal Society of Chemistry.

MXene‐based hybrids can optimize the individual properties of each component and synergistically enhance the overall properties and catalytic activity.^[^ ^201^, ^202^, ^203^ ^]^ MOFs are widely used for the preparation of hybrid materials due to their excellent structural stability and rich micro‐porous structures. A typical MOF material, Zn‐based zeolites imidazolate frameworks (ZIFs), was introduced into MXene Ti_3_C_2_T_x_ as a support for the preparation of Rh‐based catalysts (Rh/ZIF‐MX) (**Figure** **12**a).^[^ ^187^ ^]^ The incorporation of ZIF creates rich pores channels and anchor sites for Rh immobilization while preventing Ti_3_C_2_Tx nanosheets from reaggregating or repacking (Figure 12b). The ultrasmall Pd NPs are homogeneously dispersed on the surface of the hybrid support with an average particle size of only 3.2 nm, and no obvious agglomeration occurs. The Rh/ZIF‐MX catalysts demonstrated outstanding catalytic performance, including a large ECSA value (161.5 m^2^ g^−1^), high MOR catalytic activity (mass activity of 2955.1 mA mg^−1^ and specific activity 1.83 mA cm^−2^), good CO tolerance, and durable long‐term stability (Figure 12c–g). Meanwhile, the faster electron transfer rate of the Rh/ZIF‐MX catalyst is also expected to be favorable for increasing the catalytic reaction efficiency (Figure 12h). Similarly, MXene can be complex hybridized with graphene and PANI, thus facilitating the prevention of aggregation and increasing the catalyst electron transport rate.^[^ ^183^, ^184^, ^185^, ^204^ ^]^

### 4.3 Other 2D Materials

In addition to graphene and MXene mentioned above, some other 2D materials, such as g‐C_3_N_4_, GDY, layered double hydroxides (LDHs), and BPs, have attracted significant research interest in the field of supported metal catalysis in recent years. In this section, we review the recent advances in AOR from material design based on these 2D materials support. **Table** **4** shows the supported metal catalysts based on different 2D materials for AOR.

**Table 4:** Summary of supported‐metal catalysts based on different 2D material for AOR.

Catalyst	Electrolyte	ECSA [m^2^ g^−1^]	MA [mA mg^−1^]	SA [mA cm^−2^]	Stability (Retention rate)	Refs.
Pt/gCN	1.0 m CH_3_OH + 1.0 m H_2_SO_4_	68.0	310	0.46	≈78% after 300 cycles	[205]
Pt/G‐gCN	2.0 m CH_3_OH + 1.0 m H_2_SO_4_	69.0	612.8	‐	61.1% after 100 cycles	[49]
PtPd/gCN‐CB	1.0 m C_2_H_5_OH + 1.0 m NaOH	84.3	5157.0	6.11	‐	[206]
Pd/gCN‐CB	1.0 m CH_3_OH + 1.0 m NaOH	103.5	1720	1.66	‐	[207]
Pt/gCN‐CNT	1.0 m CH_3_OH + 0.5 m H_2_SO_4_	88.69	687.4	19.45	‐	[208]
Rh/G‐CN	1.0 m CH_3_OH + 1.0 m KOH	161.7	701.0	‐	40.7% after 500 cycles	[145]
Pt/NiCo‐LDH	0.5 m CH_3_OH + 0.1 m NaOH	131.86	379.2	‐	‐	[209]
Pt/IL/NiFe‐LDH	1.0 m CH_3_OH + 1.0 m NaOH	20.4	205.6	‐	97.5% after 200 cycles	[210]
Pt/C@NiRuCe‐LDH	1.0 m CH_3_OH + 1.0 m KOH	80.5	2475	‐	‐	[211]
Pd/BP‐G	1.0 m C_2_H_5_OH + 1.0 m NaOH	210.4	3960.0	‐	‐	[212]
Pd_3_P/BP	1.0 m C_2_H_5_OH + 1.0 m NaOH	462.1	7411.2	‐	‐	[213]
Pd/AG‐BP	1.0 m C_2_H_5_OH + 1.0 m NaOH	336.81	6410.87	‐	‐	[139]
Pd/ATN‐BP	1.0 m C_2_H_5_OH + 1.0 m NaOH	462.1	5023.8	‐	‐	[214]
PtCl_2_Au/GDY	1.0 m CH_3_OH + 1.0 m KOH	155.0	‐	‐	56% after 1000 cycles	[215]
Pt/NGDY	1.0 m CH_3_OH + 1.0 m KOH	69.5	1449	‐	‐	[216]
Pd/GDY	1.0 m CH_3_OH + 1.0 m KOH	144.5	3600.0	‐	95.0% after 200 cycles	[217]

#### 4.3.1 g‐C_3_N_4_

**Figure 13:**
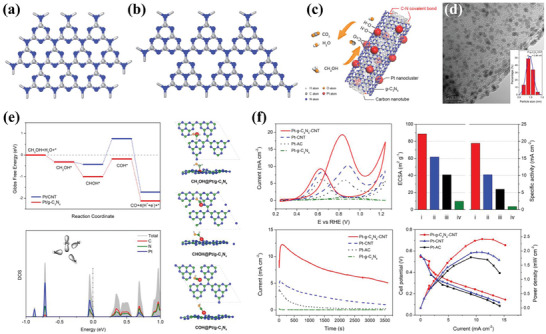
Scheme of a) *s*‐triazine and b) tri‐*s*‐triazine based connection in g‐C_3_N_4_. Blue and gray spheres represent nitrogen and carbon atoms, respectively. Reproduced with permission.^[^ ^218^ ^]^ Copyright 2012, The Royal Society of Chemistry. c) Schematic diagram of the methanol electrocatalytic processes on the Pt/g‐C_3_N_4_‐CNT catalyst. d) Typical TEM images and the Pt NP size distribution of Pt/g‐C_3_N_4_‐CNT. e) Free energy diagram; Top and side views of atomic configurations; Partial DOS plots. f) Electrocatalytic activities of Pt/g‐C_3_N_4_‐CNT composites toward MORs. Reproduced with permission.^[^ ^208^ ^]^ Copyright 2019, American Chemical Society.

g‐C_3_N_4_ is considered the most stable one of all hypothetical phases of carbon nitrides under ambient conditions, it cannot be dissolved in acidic, alkali, or organic solvents and its thermal stability up to 600 °C in air.^[^ ^218^ ^]^ Similar to graphene, g‐C_3_N_4_ possesses a stacked 2D structure, which could be regarded as a nitrogen heteroatom‐substituted graphite framework consisting of the sheet‐like π‐conjugated structure formed by C and N atoms through sp^2^ hybridization.^[^ ^219^ ^]^ As a result, it exhibits an ultra‐high nitrogen content (C/N = 0.75) with a heterocyclic macrocycle structure with N─C─N bonding pattern. The layer spacing of g‐C_3_N_4_ is ≈0.326 nm, which is slightly smaller than that of graphite, and its electrical conductivity is less than 1 S cm^−1^. As shown in **Figure** **13**a,b, There are two structural isomers of g‐C_3_N_4_, based on the s‐triazine unit (ring of C_3_N_3_) and the tri‐s‐triazine (tri‐ring of C_6_N_7_).^[^ ^220^ ^]^ The DFT calculations show that the latter is more stable than the former.^[^ ^221^ ^]^ Compared with 2D graphene, g‐C_3_N_4_ has more interesting properties, such as an ideal 2D structure, remarkable thermal and chemical stability, tunable bandgap, and easy accessibility.^[^ ^222^ ^]^ Due to these properties, g‐C_3_N_4_ has generated tremendous interest among material researchers and has the potential to be used in photocatalysis, heterogeneous catalysis, and fuel cells.^[^ ^223^, ^224^, ^225^, ^226^ ^]^ The fundamental synthesis methods for 2D g‐C_3_N_4_ nanosheets can be mainly divided into two categories: bottom‐up and top‐down approaches, through which the 2D nanosheets are prepared by directly condensing its organic precursor and exfoliating the g‐C_3_N_4_ bulk material, respectively. The precursors used in the former are usually carbon‐ and nitrogen‐rich organic small molecules, such as melamine, cyanamide, dicyandiamide, urea, thiourea, etc.^[^ ^227^ ^]^ To obtain nanosheets by exfoliation from bulk g‐C_3_N_4_, it is necessary to overcome the interlayer van der Waals forces and the hydrogen bonding of the polymeric melon units and NH/NH_2_ groups in g‐C_3_N_4_ layers. Commonly used exfoliation approaches include thermal oxidation etching, chemical exfoliation, ultrasonication‐assisted liquid phase exfoliation, and chemical vapor deposition (CVD).^[^ ^228^ ^]^ The physicochemical properties of g‐C_3_N_4_ nanosheets prepared by different synthetic methods can be obviously different, and therefore, the selection and development of suitable preparation processes according to the requirements is also crucial for their application.

Due to its nitrogen‐rich polymeric semiconductor system (C/N = 0.75), the large number of periodically separated nitrogen atoms in g‐C_3_N_4_ can effectively stabilize metal NPs through the electron lone pairs as anchor points. Therefore, g‐C_3_N_4_ can be used as a support to anchor metal NPs, which realizes the preparation of high‐performance supported metal catalysts for AORs.^[^ ^229^, ^230^ ^]^ In addition, its catalytic activity can be optimized by different strategies, including doping, functionalization and composite.

Du et al. prepared Pt/g‐C_3_N_4_ catalysts using ultrathin 2D g‐C_3_N_4_ nanosheets as supports for the deposition of ultrasmall Pt nanoclusters.^[^ ^231^ ^]^ The average size of the Pt nanoclusters was ≈3.2 nm. The prepared Pt/g‐C_3_N_4_ catalysts exhibited superior electrocatalytic performance than bare Pt NPs in MOR. Related studies have shown that 0D g‐C_3_N_4_ supported on conductive PANI not only exhibits electrocatalytic activity for methanol oxidation but also shows excellent CO tolerance to be a suitable and applicable metal free‐anode catalyst in DMFC.^[^ ^232^ ^]^ Subsequently, numerous studies have shown that the use of 2D g‐C_3_N_4_ as support for other noble metals or alloys also enhances the catalytic activity of the catalysts in AORs. For example, the supported PtRu catalysts based on g‐C_3_N_4_ show twice higher electrocatalytic activity and excellent stability for methanol oxidation due to the additional active sites from g‐C_3_N_4_.^[^ ^233^ ^]^ In addition, the use of g‐C_3_N_4_ as support also enhances the catalytic activity of non‐precious metal catalysts.^[^ ^234^, ^235^, ^236^, ^237^ ^]^ It is well known that Ni‐based has relatively good performance for MOR among many non‐precious metal catalysts. Ni NPs dispersed over 2D g‐C_3_N_4_ support were also very active electrocatalysts with MOR onset potential of 0.35 V and a current density of 57 A g^−1^.^[^ ^238^ ^]^ These results indicate that 2D g‐C_3_N_4_ nanosheets can be used as promising catalyst support in AOR.

The electronic and optical properties of g‐C_3_N_4_, regarded as a polymer semiconductor, can in principle also be modulated by elemental doping. Studies on the effect of doping g‐C_3_N_4_ with fluorine,^[^ ^239^, ^240^ ^]^ boron,^[^ ^241^ ^]^ sulfur,^[^ ^242^ ^]^ and other elements have also been reported. Doping F narrowed the bandgap of g‐C_3_N_4_ and lowered the position of the valence band. And the theoretical calculations and experimental results show that the ferromagnetism of g‐C_3_N_4_ is significantly enhanced after B doping. Elementally doped 2D g‐C_3_N_4_ materials are also widely used in HER and CO_2_ reduction reaction applications.^[^ ^243^, ^244^ ^]^ However, relevant studies on its use as support in the field of AOR have not been reported.

The performance of 2D g‐C_3_N_4_ as support in electrocatalytic AORs is still unsatisfactory due to its poor electrical conductivity. Therefore, many researches on g‐C_3_N_4_ have focused on hybridizing it in complex with other materials to obtain superior supported metal catalysts.^[^ ^145^, ^245^ ^]^ Considering that graphene also consists of lamellar packed sp^2^‐bonded carbon atoms and also has excellent electrical conductivity. Therefore, it is reasonable to combine g‐C_3_N_4_ nanosheets with graphene to generate hybrid supports, which could effectively enhance the overall electrocatalytic performance. Studies based on this composite hybridization strategy have been extensively reported in the last decade.^[^ ^246^, ^247^, ^248^, ^249^ ^]^ Due to the unique nanostructures and synergistic interactions of the two components, the Pd/g‐C_3_N_4_‐rGO catalysts prepared with its as support showed a smaller average diameter of Pd NPs (3.83 nm) and outstanding electrocatalytic properties toward MOR, far surpassing those of the Pd/rGO, Pd/g‐C_3_N_4_, and commercial Pd/C catalysts prepared through a similar approach.^[^ ^250^ ^]^ Similarly, some other carbon materials, such as CB, CNTs, and carbon nanosheets, have also been used to prepare composite support materials with g‐C_3_N_4_.^[^ ^206^, ^207^, ^251^ ^]^ Chen et al. prepared covalently coupled hybrid support (g‐C_3_N_4_‐CNT) of g‐C_3_N_4_ and CNTs for anchoring and stabilizing Pt nanoclusters (Figure 13c).^[^ ^208^ ^]^ The prepared Pt nanoclusters were uniformly and stably dispersed on the complex support with a size of only 1.0 nm (Figure 13d). The strong metal‐support interactions between g‐C_3_N_4_ and Pt clusters as well as the efficient electron transfer achieved by the overlapping of σ orbitals between g‐C_3_N_4_ and CNT, significantly enhance the catalytic performance of Pt. Calculated results show that the Pt/g‐C_3_N_4_‐CNT has a more appropriate d‐band position, significantly strengthening its adsorption behaviors for the key reaction intermediates during the methanol electro‐oxidation process and energetically decreasing the energy barriers in the multistep reaction pathways (Figure 13e). Also, the stronger binding of OH_ads_ on g‐C_3_N_4_ enables the easier removal of the poison intermediate of CO_ads_. Based on this, the as‐prepared Pt/g‐C_3_N_4_‐CNT showed improved electrocatalytic performance, such as extremely large ECSA values, excellent poison tolerances, and reliable long‐term stability, which is significantly better than commercial Pt/C and Pt/CNT (Figure 13f). A similar approach utilizing a typical conductive polymer, PANI, was reported by Ponnusamy et al.^[^ ^252^ ^]^ These attractive studies show that g‐C_3_N_4_ hybrid composites are ideal supports for loading active metal NPs.

#### 4.3.2 GDY

**Figure 14:**
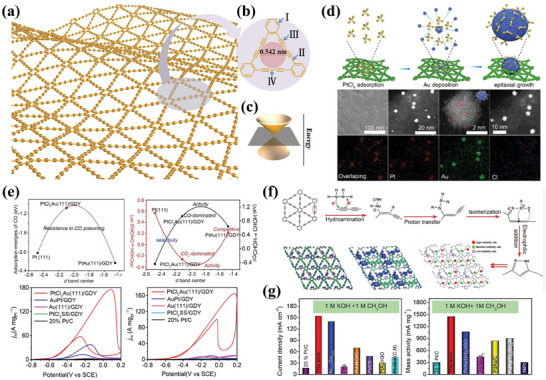
a) Schematic illustration of the basic structure of GDY. b) The corresponding pores and four types of carbon‐carbon bonding situation. c) Illustration of the Dirac cone of GDY. Reproduced with permission.^[^ ^259^ ^]^ Copyright 2019, Wiley‐VCH. d) The synthesis routes, TEM images, and EDS mapping images of the metal catalysts. e) Volcano plots of the d‐band center position of Pt, and the mass activity of the catalyst in 1 m KOH + 1 m CH_3_OH/C_2_H_5_OH. Reproduced with permission.^[^ ^215^ ^]^ Copyright 2022, American Chemical Society. f) Schematic representation of the synthetic route of the catalyst. g) Electrocatalytic performances of the catalyst for MOR. Reproduced with permission.^[^ ^216^ ^]^ Copyright 2022, Wiley‐VCH.

Graphyne is a collection of 2D carbon allotropes, which are formed by inserting acetylene linkages into graphene. This 2D carbon material containing sp^2^‐ and sp‐hybridized carbon atoms was predicted as early as 1978 by Baughman et al.^[^ ^253^ ^]^ However, it was not until 2010 that Yuliang Li et al. successfully prepared large‐area GDY films on the surface of copper,^[^ ^254^ ^]^ that they have gradually been studied and employed in energy conversion.^[^ ^255^, ^256^, ^257^ ^]^ Among all graphene, GDY was demonstrated to be the most stable carbon allotrope containing acetylene linkages.^[^ ^258^ ^]^ As shown in **Figure** **14**a,b, the fundamental structural unit of GDY consists of a coplanar sp‐sp^2^ hybridized carbon framework, a triangular ring containing 18 carbon atoms, in which each benzene ring connects with a butadiyne linkage in‐plane.^[^ ^259^ ^]^ Therefore, four types of C─C bonds exist in the GDY structure, including the C_sp2_‐C_sp2_ bonds in the benzene ring (I); the C_sp2_‐C_sp_ bonds between the benzene ring and the alkynyl group (II); the C≡C triple bonds in the alkynyl group (III); and the C_sp_‐C_sp_ single bonds connecting two adjacent alkynyl groups (IV). Owing to the existence of sp carbon, the material exhibits a special electronic structure with both Dirac points and cones (Figure 14c). These unique advantages have received remarkable attention and have shown attractive potential for use in electrocatalysis, photocatalysis, energy storage, and so on. In addition to direct synthesis on copper foils, some new methods for preparing GDY with single or only a few layers of nanosheets have been developed, including CVD coupling,^[^ ^260^ ^]^ interfacial coupling,^[^ ^261^ ^]^ and van der Waals epitaxial strategy.^[^ ^262^ ^]^

Numerous experimental and theoretical studies have shown that GDY demonstrate many unique advantages as metal catalyst support in energy storage and conversion.^[^ ^258^ ^]^ First, due to the presence of the C≡C triple bonds, GDY surface can generate high binding energy with metal NPs through chemisorption, providing uniform and abundant anchor sites for its dispersion. Unlike other 2D materials that require the construction of defects or the introduction of functional groups, some metals could be adsorbed onto the GDY surface by strong chemisorption rather than the typical physisorption.^[^ ^263^ ^]^ Moreover, the strong electronic interactions between supports and metals could prevent the migration and aggregation of metal NPs, as well as modifying electronic structure of the metal, thus enhancing the activity of the supported metal catalysts. Second, the excellent electrical conductivity and porous structure facilitates efficient electron transport and gas diffusion during electrocatalysis, which is crucial for AORs associated with long‐range charge transfer and gas generation. Third, GDY‐based materials can be prepared through a chemical method, making it easy to tune and optimize their morphology and some basic physical and chemical properties, including electrical conductivity, pore size, and affinity for metals. Also, the position and number of heteroatoms introduced into GDY can be well controlled by this preparation approach. Lastly, GDY exhibits excellent electrochemical and chemical stability, which includes has a wide stabilizing potential window and being resistant to organic solvents and strongly acidic/alkaline solutions.^[^ ^264^ ^]^ The above structural features and performance advantages make it an attractive candidate as metal catalyst supports for AORs.

The absorption of Pt clusters on graphyne, GDY, and graphene has been investigated with DFT.^[^ ^265^ ^]^ The results show that the binding energy of Pt to GDY is slightly stronger than that of graphyne, and much stronger than that of graphene, showing that the GDY and graphyne are better supports than the graphene for the stability of Pt clusters. The PtCu/GDY electrocatalysts prepared with GDY 2D nanosheets as the support exhibited excellent electrocatalytic activity for MOR with mass activity up to 336 mA mg^−1^.^[^ ^266^ ^]^ Meanwhile, the presence of the support GDY can facilitate the decomposition of H_2_O to produce OH_ads_, which significantly improves the CO poisoning tolerance of metal catalysts for MOR. In addition, the synthesis of single‐Ni‐atom‐alloyed Pt nanocrystals can be controlled by the domain‐limiting effect of porous GDY hexagonal rings.^[^ ^267^ ^]^ The prepared NiPt alloyed nanocrystals with uniform size of only 2.18 nm exhibited the greatly enhancement in the durability and activity in MOR. These results demonstrate the feasibility of GDY‐based for the design of high‐performance supported metal catalysts for AORs.

This catalyst with GDY as metal support has been extensively used in AORs. Yuliang Li et al. prepared multicomponent quantum dots with a size of 2.37 nm by epitaxial growth of Au quantum dots using atomically Pt chlorine species with porous GDY as a support (Figure 14d).^[^ ^215^ ^]^ The GDY supports offer ideal support for in situ confinement growth of metal atoms and quantum dots. Meanwhile, chlorine tuned the d‐band structure of the Pt surface and suppressed the CO poisoning pathway of MOR and EOR (Figure 14e). Through these rational designs, the as‐prepared supported metal catalysts exhibited excellent catalytic performance, show high mass activity of 175.64 A mg_pt_^−1^ and 165.35 A mg_pt_^−1^ in MORs and EORs, respectively (Figure 14e). Meanwhile, the catalysts also exhibit long‐lasting stability for MORs. Furthermore, they also reported a method to control the structure and performance of catalysts by atomic arrangement.^[^ ^217^ ^]^ In this work, they synthesized 3D urchin‐like Pd NPs with defect‐rich structure, low coordination number (CN), and tensile strain by introducing chlorine into the GDY supports, which enabled the modulation of the arrangement of metal atoms in Pd NPs. The test results showed that the prepared catalysts were beneficial to the oxidation and removal of CO intermediates, and exhibited excellent stability for MOR, maintaining 95% activity after 2000 cycles. Moreover, the catalysts exhibited high current density (363.6 mA cm^−2^) and mass activity (3.6 A mg_pa_^−1^).

The GDY, which can be synthesized through a chemical method and consist of sp and sp^2^ hybridized carbon atoms, provide favorable platform for rational doping of elements. Heteroatom‐doped GDY has stronger chemical bonding and more stable anchor sites for metal atoms due to its defect engineering. Moreover, the electronegativity of the heteroatoms induces more charge transfer between the GDY support and the metal, which results in more stable and active metal catalyst on the GDY support. This endows GDY with special properties and have been widely used in various applications such as catalysis, energy storage and conversion, and provides an opportunity for the preparation of high‐performance supported metal catalysts for AORs.^[^ ^268^ ^]^ The N‐doped GDYs obtained by selective cycloaddition of sp‐hybridized carbon atoms in GDY with hydrazine by Yuliang Li et al (Figure 14f).^[^ ^216^ ^]^ It is used as a support to realize anchoring of zero‐valent Pt atoms. The prepared Pt atomic catalysts exhibit excellent activity, high pH suitability, and high CO tolerance for MOR (Figure 14g). Theoretical calculations show that this excellent catalytic performance was attributed to the high coverage and dispersion of Pt atoms due to the incorporation of highly electronegative N atoms, which enhances the fixation of intermediates and electron transfer for MOR. In addition to N atoms, other atoms, such as B, Cl, F, etc., can also be introduced into GDY to improve its electronic properties and catalytic activity in certain applications.^[^ ^269^, ^270^, ^271^ ^]^ For instance, the introduction of Cl atoms into GDY improves the activity, selectivity, and stability of the nitrogen reduction reactions.^[^ ^268^ ^]^ However, there are fewer reports on other heteroatom‐doped GDYs as supports for metal catalysts in the field of AORs.

#### 4.3.3 LDHs

**Figure 15:**
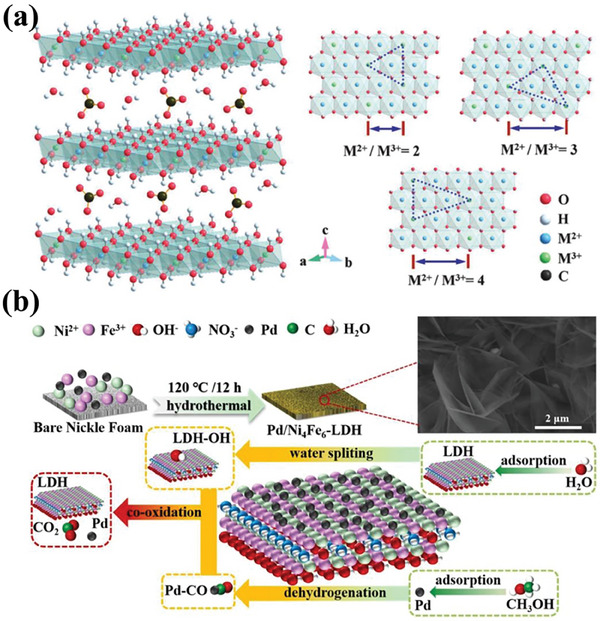
a) Lamellar LDH structure with different metal cations and intercalated anions. Reproduced with permission.^[^ ^272^ ^]^ Copyright 2014, The Royal Society of Chemistry. b) Synthesis route and reaction mechanism diagram of Pd/NiFe‐LDH. Reproduced with permission.^[^ ^286^ ^]^ Copyright 2023, Elsevier.

LDHs are a class of 2D brucite‐like layered clay materials composed of bivalent and/or trivalent cations, which have the advantages of being geologically abundant, inexpensive, and low toxicity. As seen in **Figure** **15**a, the formula is expressed as [M_1−x_^2+^M_x_^3+^(OH)_2_]^x+^(A_x/n_)^n−^·mH_2_O, where M^2+^ and M^3+^ represent bivalent (e.g., Mg^2+^, Ni^2+^, Co^2+^, Zn^2+^, Cu^2+^) and trivalent (Al^3+^, Cr^3+^, Mn^3+^, In^3+^, Ga^3+^) transition metal cations, respectively, and A^n−^ is the interlayer anion.^[^ ^272^ ^]^ Considering the structural stability, the value of x is usually in the range of 0.2 and 0.4.^[^ ^273^, ^274^ ^]^ Therefore, the adjustment of interlayer spacing and modulation of electronic structure can be achieved by regulating the transition metal cations and anions in LDHs. Synthesis strategies for fabricating 2D LDH nanosheets can generally be classified into top‐down and bottom‐up strategies.^[^ ^275^ ^]^ Due to their layered structure and diverse compositions, LDH‐based materials have become a research hot in the field of electrocatalysis and have been widely used in various energy conversion applications such as OER and HER.^[^ ^276^, ^277^ ^]^ Of course, this opens up the possibility of its use as a catalyst and catalyst support for AOR.

Due to the abundance of non‐precious metal active sites on LDHs nanosheets, they were directly used as catalysts for AOR in replacement of noble metal‐based catalysts. For instance, pristine LDH nanosheets such as NiCo‐LDHs and NiFe‐LDHs exhibit good catalytic activity toward MOR and EOR, the abundance of Ni(III) active species.^[^ ^278^, ^279^, ^280^ ^]^ By further modulating the electronic configuration of LDH nanosheets, their adsorption capacity for methanol and ethanol could be optimized, which enhanced their intrinsic MOR and EOR activities.^[^ ^281^, ^282^ ^]^

Except for their direct use as catalysts, the use of LDHs as catalyst support is another mainstream research field for the preparation of highly active AOR catalysts. Li et al. investigated in detail the electrocatalytic activity of MgAl‐LDH‐loaded Au NPs catalysts for methanol oxidation in alkaline media.^[^ ^283^ ^]^ Under the same conditions, the electrocatalytic activity of Au NPs/MgAl‐LDH catalysts was higher than that of pure AuNPs and MgAl‐LDH. The promoting effect of LDHs could be due to their strong adsorption to OH^−^ and the discharge of OH^−^ during methanol oxidation. Prevot et al. reported a one‐step preparation of AuNP/LDH‐supported metal catalysts via the polyol route. Due to the synergistic effect between Ni and Au NPs sites, the highest 4‐fold catalytic efficiency observed in AuNP/LDH catalysts compared to pristine NiAl‐LDH was observed for methanol oxidation.^[^ ^284^ ^]^ Some other transition metal cationic LDHs, such as NiFe, CoFe, and CoAl, have also been widely used as support, thereby enhancing the catalytic performance of the catalysts for AOR.^[^ ^210^, ^211^, ^285^ ^]^ Similarly, 2D LDHs as a support are also beneficial for enhancing the electrocatalytic activity of noble metal‐based catalysts for AOR. Xiaoqiang Wu and co‐authors reported that LDH support could inhibit CO‐poisoning by the precious metal Pd (Figure 15b).^[^ ^286^ ^]^ In this work, the supported metal catalyst Pd/NiFe‐LDH was prepared via the hydrothermal method. NiFe‐LDH not only provides a large surface for Pd loading but also provides an effective platform for the formation of OH_ads_, which promotes the oxidation of CO_ads_ (2OH_ads_ + CO_ads_ → CO_2_ + H_2_O), and thus improves the activity of Pd. As a result, the as‐prepared Pd/NiFe‐LDH exhibits high electrocatalytic activity (2665.3 mA mg_Pd_^−1^) and abundant ECSA (110.75 cm^2^ mg^−1^). Furthermore, Pd/NiFe‐LDH maintained an excellent mass activity of 16.1 mA mg_Pd_^−1^ after 40 h of i‐t operation. Huang et al. reported that MgAl‐LDH/Pt shows more tolerance to CO poisoning due to its greater tendency to generate hydroxyl radicals rather than the OER, which commonly occurs on NiFe‐LDH surfaces.^[^ ^287^ ^]^ These findings suggest that the introduction of 2D atomic layers of LDH sheet removes poisonous carbonaceous intermediates from metal surfaces, thereby improving the catalytic activity and long‐term stability for AOR.

However, several drawbacks of LDHs, such as low electrical conductance, insufficient active sites, and stability during long‐term electrolysis, have greatly hindered their application as catalysts or support in electrochemical catalysis. Because of the lack of interlayer anions, 2D LDHs nanosheets with positive charge can easily hybridize with negatively charged nanostructured materials (e.g., graphene, CNTs, and metal oxides), which is conducive to alleviating the agglomeration of the LDHs nanosheets, increasing the overall electrical conductivity, and ameliorating the electronic structure, which leads to the optimization of electrocatalytic performance.^[^ ^288^, ^289^ ^]^ For example, the prepared Pt/NiFe‐LDH/rGO electrocatalysts exhibited higher peak current density (949.3 mA mg^−1^), larger ECSA (24.6 m^2^ g_Pt_^−1^), and better stability for methanol oxidation compared to its rGO‐free counterpart (512.3 mA mg^−1^ and 17.7 m^2^ g_Pt_^−1^).^[^ ^290^ ^]^ Therefore, LDH hybridized composite nanomaterials have attracted much attention and have emerged as highly conductive support materials for electrocatalytic applications for AOR.

#### 4.3.4 BPs

**Figure 16:**
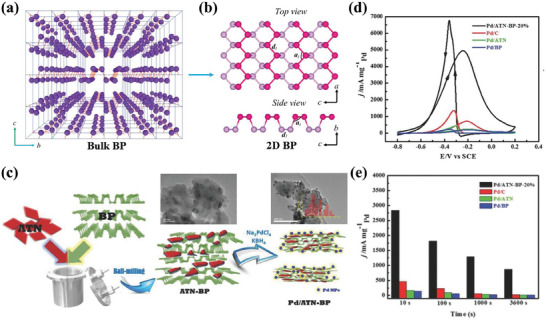
a) Atomic model of bulk BP. Reproduced with permission.^[^ ^301^ ^]^ Copyright 2016, Nature. b) Atomic models of 2D BP. Reproduced with permission.^[^ ^302^ ^]^ Copyright 2009, American Chemical Society. c) Schematic illustration of the fabrication of the Pd/ATN‐BP hybrids and TEM images of the Pd/ATN‐BP hybrids. d) Cyclic voltammetry of different catalysts. e) Comparison of the current densities for the EOR after durability tests with different durations. Reproduced with permission.^[^ ^214^ ^]^ Copyright 2017, Wiley‐VCH.

BPs with a 2D layered structure is the most stable allotrope of phosphorus, which was first prepared under high pressure in 1914.^[^ ^291^ ^]^ Up until 2014, when it was used as a 2D semiconductor material for field‐effect transistors, its unique structure and properties, such as high charge‐carrier mobility (1000 cm^2^ V^−1^ s^−1^ at room temperature), distinct pleated structures, and interesting in‐layer anisotropies attracted much attention from scientists (**Figure** **16**a,b).^[^ ^292^, ^293^ ^]^ BP has many potential applications in the field of catalysis, showing excellent electrocatalytic activity as an electrocatalyst in OER and HER.^[^ ^294^, ^295^, ^296^ ^]^ Meanwhile, it can be used as support to anchor metal NPs such as Pt, Pd.^[^ ^297^, ^298^ ^]^ It is worth noting that each phosphorus atom in each layer of BP possesses a lone pair of electrons. When used as support, this may lead to electron transfer between the supported metal and the BP, thus changing the d‐band center of the active metal. Preparation methods for 2D BPs include mechanical exfoliation, electrochemical assistance, and liquid exfoliation.

Peruzzini et al. demonstrated strong interactions between metal Pd and 2D BP support using electron energy loss spectroscopy‐scanning transmission electron microscopy.^[^ ^299^ ^]^ Quantitative characterization of this interaction using X‐ray absorption spectroscopy shows that Pd‐P exhibits a short distance of only 2.26 Å, indicating that the interaction is a Pd‐P coordination bond of covalent nature. The formation of Pd‐P bonds produces a strong synergistic ligand effect on Pt NPs and changes the coordination environment of Pd atoms. At the same time, the electronic structure of metal NPs in supported‐metal catalysts significantly affects the adsorption energy and activation barrier about interfacial reactants or intermediates in electrochemical catalysis. Similarly, Pt‐based catalysts based on BP were investigated by Yu et al.^[^ ^300^ ^]^ The results indicate that BP has a strong activation effect on Pt catalysts through the synergistic ligand effect between metal and support. The Pt‐P bonding down‐shifts the d‐band center of Pt toward the Fermi level, thus providing optimal energy for the intermediates of the electrocatalytic HER. These attractive studies show that 2D BPs can also be used as support for metal NPs for the preparation of highly active catalysts for AOR.

There are several advantages to using 2D BP as metal catalyst support for AOR: i) As a 2D layered structure material, BP has a large surface area, which is beneficial to improve the dispersion of metal NPs and increase the number of active sites, resulting in improved utilization of metal catalysts. ii) The presence of lone pairs of electrons in BP readily coordinates with metal atoms to produce strong interactions and changes the d‐band centers of the catalyst metal atoms to increase the reactivity. iii) BP has an excellent adsorption capacity for H_2_O, thus promoting the generation of OH_ads_ and the stripping of reaction intermediates CO_ads_. iv) The strong coordination bond formed between BP and metal prevents metal detachment and agglomeration during the reaction process and improves the long‐term stability of the catalyst. However, the BP nanosheets are chemically unstable and easily oxidized, which greatly restricts their application as 2D material support.^[^ ^303^ ^]^ Surface modification or doping is a convenient method to inhibit the oxidative degradation of 2D BP.^[^ ^304^ ^]^ For example, tannin (TA) or polyurethane functionalized BP nanosheets show improved stability when exposed to oxygen and water for an extended time.^[^ ^305^, ^306^ ^]^ However, the modifying groups or doping elements would coordinate with the lone electrons of the P atoms, damaging their inherent structural advantages as catalyst support. Therefore, the development of processing techniques for the preparation of stable, large‐area, high‐performance 2D BP nanosheets is particularly critical for their further development.

Preparation of BP‐based heterostructures by hybridization composite strategy can improve the catalytic performance of supported‐metal catalysts for AORs.^[^ ^139^, ^307^ ^]^ In recent years, many related studies have been continuously carried out. For instance, Yulin Min, Qunjie Xu, and coauthors prepared BP‐ATN hybrids interconnected by P─O─Ti bonds by direct ball‐milling of BP nanoflakes and anatase titanium dioxide (ATN) under argon protection (Figure 16c).^[^ ^214^ ^]^ Then, supported‐metal catalysts for EOR were fabricated using the hybrids as supports for Pd NPs. This well‐designed heterostructure of hybrids greatly increases the catalytic activity for EOR with the high electrochemically active area (462.1 m^2^ g_Pd_^−1^) and mass activity (5023.8 mA mgPd^−1^) due to the high synergistic interaction between the Pd NPs and ATN‐BP and the structural contribution (Figure 16d,e). Similar approaches were also reported by Jun Xu et al.^[^ ^213^ ^]^ and Mengkui Tian et al.^[^ ^212^ ^]^ The design of heterostructured materials makes efficient catalysis, demonstrating the potential of BP‐based materials as metal support.

In addition, there are also 2D materials such as chalcogenides, 2D conductive polymers, and 2D MOFs or covalent organic frameworks (COFs) that can be used as support in metal catalysts.^[^ ^308^, ^309^, ^310^, ^311^, ^312^ ^]^ A classic example was the novel metal catalyst for AOR prepared with chalcogenides as support. The chalcogenides of the general formula MX_2_ include a large family of compounds, where M is a 4B, 5B, or 6B transition metal and X is S, Se, or Te, such as MoS_2_, VS_2_, NbSe_2_, and WTe_2_. As a prototypical compound of chalcogenides, MoS_2_ has been widely investigated as catalyst and optoelectronic material.^[^ ^313^ ^]^ The Pd‐MoS_2_ metal catalysts prepared with it as a support have enhanced catalytic activity with 2.8‐fold anodic peak current mass density compared to a commercial Pd/C catalyst, which suggests its potential application in DMFC.^[^ ^314^ ^]^ Preparation of 3D structures by composite hybridization can reduce the *π*–*π* stacking of MoS_2_ nanosheets, which is favorable for the dispersion of metal NPs, and promoting the electron transport on the MoS_2_ substrate surface, thus improving the activity and stability of metal catalysts. This strategy has been widely employed for the preparation of high‐performance supported metal catalysts for AORs by compositing MoS_2_ with graphene.^[^ ^143^, ^144^ ^]^ In addition, MoS_2_ can also be used to prepare photoassistant support by constructing 2D heterojunctions due to the narrow bandgap (1.2–1.9 eV). The synergistic effect of photo‐ and electro‐catalytic in heterojunction facilitates the catalytic performance of AOR.^[^ ^315^ ^]^

## 5 Summary and Perspective

**Figure 17:**
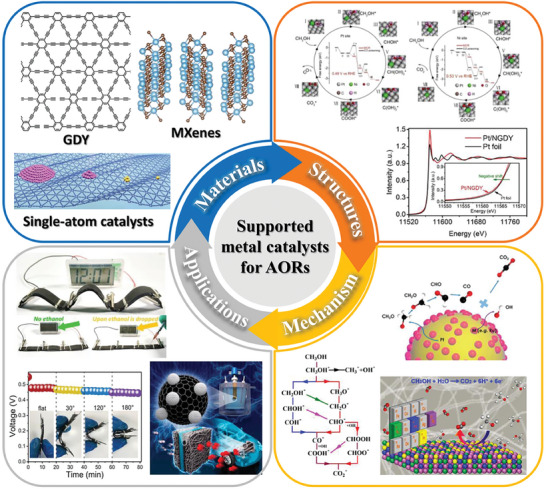
The perspectives on future material design, structural characterization, and mechanism investigation for the application of 2D supported metal catalyst materials. Reproduced with permission.^[^ ^26^, ^216^, ^267^, ^316^, ^317^, ^318^, ^319^, ^320^, ^321^ ^]^ Copyright 2017 and 2021, American Chemical Society; Copyright 2019, 2021, and 2022, Elsevier; Copyright 2020, 2021, and 2022, Wiley‐VCH; Copyright 2022, Springer.

As a breakthrough in the development of electrocatalysts for AOR, 2D layer materials (such as graphene, MXene, g‐C_3_N_4_, GDY, LDH, and BP) have been demonstrated as efficient catalyst supports due to their large surface areas, high dispersion of active metal components, and good electrical conductivity. Particularly, the modification strategies have been developed to tune 2D material supports, including surface functionalization modifications, heteroatom doping, and composite hybridization, which could enhance metal dispersion, minizine reaction energy barriers, and enhance electrocatalytic stability. Nevertheless, 2D material supports are still facing some challenges, which require future efforts to find their solutions (**Figure** **17**):

i) Materials: We discussed the material aspects in detail in this review. Although, rich material selectivity has been realized now in the field of AORs. However, optimizing or designing new materials is still the most popular and fundamental approach for improving the overall catalytic performance of AORs. On the one hand, further development of novel 2D support materials is required. Due to the many unique structural advantages of different 2D supports, active metals structure can be effectively modulated and optimized by developing new supports. For example, The sp‐C in 2D GDY provides a specific site for anchoring metal atoms or nanoclusters and serves as an active site for improving catalytic performance. On the other hand, single‐atom catalysts (SACs) have attracted intensive attention due to their excellent catalytic performance and nearly 100% atom utilization for numerous processes.^[^ ^322^, ^323^, ^324^, ^325^ ^]^ However, a single Pt atom dispersed on carbon support could not catalyze AOR, because three contiguous Pt atoms on the carbon support are required to form the smallest group of atoms that can oxidize methanol to CO_2_.^[^ ^170^, ^326^, ^327^ ^]^ However, when the Pt‐O_3f_‐Ru_cus_ with O_br_ was formed in Pt_1_/RuO_2_, Pt single atoms became active for MOR.^[^ ^328^ ^]^ Single Pt atoms on Ru NPs also showed MOR catalytic activity.^[^ ^329^ ^]^ Furthermore, these SACs exhibit much higher catalytic activity than Pt/C. These interesting findings not only demonstrate the influence of atomic coordination environment on catalytic performance but also indicates the promising of SACs for AOR. Therefore, Single Pt (or other noble metals) atoms on 2D non‐carbon supports would deserve special attention.
ii) Structures: The variety in the selection of 2D materials support provides abundant opportunities for tailoring chemical and physical properties, but this also causes many difficulties in identifying the catalyst structure, especially the interactions between the support and the metal. This will inspire ones to achieve comprehensive qualitative characterization of catalyst structures by combining various techniques, such as X‐ray absorption near‐edge structure (XANES), X‐ray photoelectron spectroscopy (XPS), Raman spectroscopy and Fourier transform infrared spectroscopy (FTIR), creating the correlations between the structures and catalytic performance of catalysts and thus providing a guide for the design of highly efficient 2D layer material‐based catalysts.
iii) Mechanism: One of the biggest challenges in developing high‐performance AOR catalysts is the lack of comprehensive understanding of the catalytic mechanism and process. The reaction pathways and RDS of AOR over 2D material‐based catalysts have not yet been clearly identified. Furthermore, the complicated surface and interfacial chemistry result in additional challenges in understanding the involved chemical reactions at molecular level. To solve these issues, the in situ characterization under real catalytic conditions should be developed, particularly by monitoring the intricate chemical transformation and the evolution of active sites. For example, in situ liquid phase‐TEM with in situ micro‐spectroscopic techniques, online electrochemical mass spectrometry, and in situ electron paramagnetic resonance would allow to observe the structural evolution of the active sites as well as the chemical reactions of the adsorbed. Moreover, supported metal catalysts exhibited more complex active sites compared to metal alloys. Therefore, it is crucial to overcome the limitations of DFT calculations and accurately model the catalysts. In addition, the effect of the catalytic environment such as anions and cations adsorbed on the material surface, and the potential on the reaction process needs to be carefully considered to ensure the reliability of the calculation results.
iv) Applications: Better application of the prepared high‐performance carrier metal catalysts to real‐life applications is the ultimate goal of the research. First, novel practical devices can be developed for electrocatalysts for AORs. For example, Huang et al. reported flexible drop‐and‐play direct ethanol fuel cells with excellent flexibility and unique drop‐and‐play function that can easily power electronic clocks and smartphones.^[^ ^318^, ^319^ ^]^ Second, another important aspect to consider is to ensure that the materials used to synthesize the catalysts are economical. Current processes for the synthesis of 2D material‐based catalysts for AOR are restricted to the lab‐scale. For wide applications of these catalysts in industry, they must be produced at a large scale in cost‐effective way without sacrificing catalytic performance, which should attract attention particularly from industry. Lastly, in practical devices, the operating conditions are very different and more factors affect activity and stability of catalysts than in lab‐scale tests. It is crucial to establish new standards and evaluation directions to link lab research with practical applications.

## Conflict of Interest

The authors declare no conflict of interest.

## References

[1] L. Yang , J. Shui , L. Du , Y. Shao , J. Liu , L. Dai , Z. Hu , Adv. Mater. 2019, 31, 1804799. 10.1002/adma.201804799 30637835

[2] Z. Lin , Z. Wang , J. Gong , T. Jin , S. Shen , Q. Zhang , J. Wang , W. Zhong , Adv. Funct. Mater. 2023, 33, 2112832.

[3] H. Su , Y. H. Hu , Chem. Eng. J. 2020, 402, 126235.

[4] H. Sua , W. Zhanga , Y. H. Hua , Proc. Natl. Acad. Sci. USA 2022, 119, 2208750119.

[5] M. A. Ud Din , M. Idrees , S. Jamil , S. Irfan , G. Nazir , M. A. Mudassir , M. S. Saleem , S. Batool , N. Cheng , R. Saidur , J. Energy Chem. 2023, 77, 499.

[6] H. Kim , T. Y. Yoo , M. S. Bootharaju , J. H. Kim , D. Y. Chung , T. Hyeon , Adv. Sci. 2022, 9, 2104054. 10.1002/advs.202104054 PMC8728832 34791823

[7] W. Qiao , X. Huang , L. Feng , Chin. J. Struct. Chem. 2022, 41, 2207016.

[8] F. Lyu , M. Cao , A. Mahsud , Q. Zhang , J. Mater. Chem. A 2020, 8, 15445.

[9] W. Fang , L. Huang , S. Zaman , Z. Wang , Y. Han , B. Y. Xia , Chem. Res. Chin. Univ. 2020, 36, 611.

[10] H. Wang , L. Jiao , L. Zheng , Q. Fang , Y. Qin , X. Luo , X. Wei , L. Hu , W. Gu , J. Wen , C. Zhu , Adv. Funct. Mater. 2021, 31, 2103465.

[11] T. Xia , K. Zhao , Y. Zhu , X. Bai , H. Gao , Z. Wang , Y. Gong , M. Feng , S. Li , Q. Zheng , S. Wang , R. Wang , H. Guo , Adv. Mater. 2023, 35, 2206508. 10.1002/adma.202206508 36281798

[12] M. Du , X. Li , H. Pang , Q. Xu , EnergyChem 2023, 5, 100083.

[13] Z. Wang , Z. Lin , Y. Wang , S. Shen , Q. Zhang , J. Wang , W. Zhong , Adv. Mater. 2023, 35, 2302007. 10.1002/adma.202302007 36994807

[14] Z. Wang , B. Xiao , Z. Lin , S. Shen , A. Xu , Z. Du , Y. Chen , W. Zhong , J. Energy Chem. 2021, 54, 510.

[15] A. J. Medford , A. Vojvodic , J. S. Hummelshøj , J. Voss , F. Abild‐Pedersen , F. Studt , T. Bligaard , A. Nilsson , J. K. Nørskov , J. Catal. 2015, 328, 36.

[16] Z.‐J. Zhao , S. Liu , S. Zha , D. Cheng , F. Studt , G. Henkelman , J. Gong , Nat. Rev. Mater. 2019, 4, 792.

[17] X. Chia , M. Pumera , Nat. Catal. 2018, 1, 909.

[18] R. Gusmão , M. Veselý , Z. Sofer , ACS Catal. 2020, 10, 9634.

[19] W. J. Niu , J. Z. He , B. N. Gu , M. C. Liu , Y. L. Chueh , Adv. Funct. Mater. 2021, 31, 2103558.

[20] L. X. Chen , Z. W. Chen , M. Jiang , Z. Lu , C. Gao , G. Cai , C. V. Singh , J. Mater. Chem. A 2021, 9, 2018.

[21] C. Yan , Y. L. Liu , Q. Zeng , G. G. Wang , J. C. Han , Adv. Funct. Mater. 2022, 33, 2210837.

[22] J. Yang , W. Li , D. Wang , Y. Li , Adv. Mater. 2020, 32, 2003300. 10.1002/adma.202003300 33125802

[23] Q. Q. Yan , P. Yin , H. W. Liang , ACS Mater. Lett. 2021, 3, 1197.

[24] X. Zhao , Q. Liu , Q. Li , L. Chen , L. Mao , H. Wang , S. Chen , Chem. Eng. J. 2020, 400, 125744.

[25] C. Lamy , A. Lima , V. LeRhun , F. Delime , C. Coutanceau , J.‐M. LeÂger , J. Power Sources 2002, 105, 283.

[26] Y. Tong , X. Yan , J. Liang , S. X. Dou , Small 2021, 17, 1904126. 10.1002/smll.201904126 31608601

[27] R. Zeng , Y. Yang , T. Shen , H. Wang , Y. Xiong , J. Zhu , D. Wang , H. D. Abruña , ACS Catal. 2019, 10, 770.

[28] Z. Qi , C. Xiao , C. Liu , T. W. Goh , L. Zhou , R. Maligal‐Ganesh , Y. Pei , X. Li , L. A. Curtiss , W. Huang , J. Am. Chem. Soc. 2017, 139, 4762. 28272879 10.1021/jacs.6b12780

[29] G. A. Tritsaris , J. Rossmeisl , J. Phys. Chem. C 2012, 116, 11980.

[30] S. Bai , Y. Xu , K. Cao , X. Huang , Adv. Mater. 2021, 33, 2005767. 10.1002/adma.202005767 33314444

[31] Y. Qin , H. Huang , W. Yu , H. Zhang , Z. Li , Z. Wang , J. Lai , L. Wang , S. Feng , Adv. Sci. 2022, 9, 2103722. 10.1002/advs.202103722 PMC8844492 34951154

[32] J. Li , L. Li , X. Ma , X. Han , C. Xing , X. Qi , R. He , J. Arbiol , H. Pan , J. Zhao , J. Deng , Y. Zhang , Y. Yang , A. Cabot , Adv. Sci. 2023, 10, 2300841. 10.1002/advs.202300841 PMC10214232 36950758

[33] Y. Wang , J. Liu , H. Yuan , F. Liu , T. Hu , B. Yang , Adv. Funct. Mater. 2023, 33, 2211909.

[34] G. M. Schwab , J. Colloid Interface Sci. 1970, 34, 337.

[35] C. T. Campbell , Nat. Chem. 2012, 4, 597. 22824888 10.1038/nchem.1412

[36] Z. W. Seh , J. Kibsgaard , C. F. Dickens , I. Chorkendorff , J. K. Norskov , T. F. Jaramillo , Science 2017, 355, eaad4998. 28082532 10.1126/science.aad4998

[37] S. Jiao , X. Fu , H. Huang , Adv. Funct. Mater. 2021, 32, 2107651.

[38] W. Meng , H. He , L. Yang , Q. Jiang , B. Yuliarto , Y. Yamauchi , X. Xu , H. Huang , Chem. Eng. J. 2022, 450, 137932.

[39] D. Xiao , Q. Jiang , C. Xu , C. Yang , L. Yang , H. He , H. Huang , J. Colloid Interface Sci. 2022, 616, 781. 35247815 10.1016/j.jcis.2022.02.111

[40] S. Li , X. Liang , S. Shen , H. Yang , C. L. Wu , Inorg. Chem. 2021, 60, 17388. 34709791 10.1021/acs.inorgchem.1c02953

[41] J. Cao , X. Wu , Y. Chen , Z. Chen , R. Jiang , Y. Zhao , Q. Tian , D. Chen , S. Xiao , B. Yu , Y. Jin , J. Alloys Compd. 2023, 955, 170240.

[42] H. Yang , S. Li , F. Feng , S. Ou , F. Li , M. Yang , K. Qian , J. Jin , J. Ma , ACS Sustainable Chem. Eng. 2019, 7, 14621.

[43] Y. Sun , H. Zheng , C. Wang , M. Yang , A. Zhou , H. Duan , Nanoscale 2016, 8, 1523. 26681401 10.1039/c5nr06912b

[44] Y. Jiao , Y. Zheng , M. Jaroniec , S. Z. Qiao , Chem. Soc. Rev. 2015, 44, 2060. 25672249 10.1039/c4cs00470a

[45] Z. Chen , J. Cao , X. Wu , D. Cai , M. Luo , S. Xing , X. Wen , Y. Chen , Y. Jin , D. Chen , Y. Cao , L. Wang , X. Xiong , B. Yu , ACS Appl. Mater. Interfaces 2022, 14, 12223. 35235300 10.1021/acsami.1c23718

[46] S. K. Venishetty , S. Kummari , S. Karingula , S. Moru , K. V. Gobi , Int. J. Hydrogen Energy 2023, 48, 21487.

[47] C. Yang , Q. Jiang , W. Li , H. He , L. Yang , Z. Lu , H. Huang , Chem. Mater. 2019, 31, 9277.

[48] H. Yan , M. Meng , L. Wang , A. Wu , C. Tian , L. Zhao , H. Fu , Nano Res. 2016, 9, 329.

[49] H. Huang , S. Yang , R. Vajtai , X. Wang , P. M. Ajayan , Adv. Mater. 2014, 26, 5160. 24956285 10.1002/adma.201401877

[50] J. Bai , N. Jia , P. Jin , P. Chen , J.‐X. Jiang , J.‐H. Zeng , Y. Chen , J. Energy Chem. 2020, 51, 105.

[51] R. Arukula , M. Vinothkannan , A. R. Kim , D. J. Yoo , J. Alloys Compd. 2019, 771, 477.

[52] K. Zhang , Y. Hu , L. Wang , M. J. Monteiro , Z. Jia , ACS Appl. Mater. Interfaces 2017, 9, 34900. 28956591 10.1021/acsami.7b09604

[53] Y. Shao , Z. Zha , H. Wang , J. Energy Chem. 2021, 63, 54.

[54] J. Ma , A. Habrioux , Y. Luo , G. Ramos‐Sanchez , L. Calvillo , G. Granozzi , P. B. Balbuena , N. Alonso‐Vante , J. Mater. Chem. A 2015, 3, 11891.

[55] H. Cui , Y. Guo , L. Guo , L. Wang , Z. Zhou , Z. Peng , J. Mater. Chem. A 2018, 6, 18782.

[56] W. Chen , M. Wan , Q. Liu , X. Xiong , F. Yu , Y. Huang , Small Methods 2018, 3, 1800323.

[57] X. Feng , Y. Bai , M. Liu , Y. Li , H. Yang , X. Wang , C. Wu , Energy Environ. Sci. 2021, 14, 2036.

[58] Y. Han , X. Yan , Q. Wu , H. Xu , Q. Li , A. Du , X. Yao , Small Struct. 2023, 4, 2300036.

[59] N. Logeshwaran , I. R. Panneerselvam , S. Ramakrishnan , R. S. Kumar , A. R. Kim , Y. Wang , D. J. Yoo , Adv. Sci. 2022, 9, 2105344. 10.1002/advs.202105344 PMC8922112 35048552

[60] Q. Sun , X.‐H. Li , K.‐X. Wang , T.‐N. Ye , J.‐S. Chen , Energy Environ. Sci. 2023, 16, 1838.

[61] W. Kong , J. Deng , L. Li , J. Mater. Chem. A 2022, 10, 14674.

[62] H. F. Wang , C. Tang , C. X. Zhao , J. Q. Huang , Q. Zhang , Adv. Funct. Mater. 2022, 32, 2204755.

[63] Y. Xiao , Y. X. Pang , Y. Yan , P. Qian , H. Zhao , S. Manickam , T. Wu , C. H. Pang , Adv. Sci. 2023, 10, 2205292. 10.1002/advs.202205292 PMC10037997 36658693

[64] Q. T. Lai , X. H. Zhao , Q. J. Sun , Z. Tang , X. G. Tang , V. A. L. Roy , Small 2023, 19, 2300283. 10.1002/smll.202300283 36965088

[65] K. S. Novoselov , A. K. Geim , S. V. Morozov , D. Jiang , Y. Zhang , S. V. Dubonos , I. V. Grigorieva , A. A. Firsov , Science 2004, 306, 666. 15499015 10.1126/science.1102896

[66] G. Kothandam , G. Singh , X. Guan , J. M. Lee , K. Ramadass , S. Joseph , M. Benzigar , A. Karakoti , J. Yi , P. Kumar , A. Vinu , Adv. Sci. 2023, 10, 2301045. 10.1002/advs.202301045 PMC10288283 37096838

[67] W. Zhu , Y. Pei , H. Liu , R. Yue , S. Ling , J. Zhang , X. Liu , Y. Yin , M. D. Guiver , Adv. Sci. 2023, 10, 2206062. 10.1002/advs.202206062 PMC10323636 37162215

[68] F. Pan , B. Li , E. Sarnello , Y. Fei , X. Feng , Y. Gang , X. Xiang , L. Fang , T. Li , Y. H. Hu , G. Wang , Y. Li , ACS Catal. 2020, 10, 10803.

[69] Z. Sun , Y. H. Hu , Acc. Mater. Res. 2020, 2, 48.

[70] Z. Sun , S. Fang , Y. H. Hu , Chem. Rev. 2020, 120, 10336. 32852197 10.1021/acs.chemrev.0c00083

[71] S. Gilje , S. Han , M. Wang , K. L. Wang , R. B. Kaner , Nano Lett. 2007, 7, 3394. 17944523 10.1021/nl0717715

[72] X. Yu , H. Cheng , M. Zhang , Y. Zhao , L. Qu , G. Shi , Nat. Rev. Mater. 2017, 2, 17046.

[73] I. Fampiou , A. Ramasubramaniam , J. Phys. Chem. C 2012, 116, 6543.

[74] E. Yoo , T. Okata , T. Akita , M. Kohyama , J. Nakamura , I. Honma , Nano Lett. 2009, 9, 2255. 19405511 10.1021/nl900397t

[75] S. Sharma , A. Ganguly , P. Papakonstantinou , X. Miao , M. Li , J. L. Hutchison , M. Delichatsios , S. Ukleja , J. Phys. Chem. C 2010, 114, 19459.

[76] J.‐J. Shao , Z.‐J. Li , C. Zhang , L.‐F. Zhang , Q.‐H. Yang , J. Mater. Chem. A 2014, 2, 1940.

[77] Y. Kang , Q. Xue , P. Jin , J. Jiang , J. Zeng , Y. Chen , ACS Sustainable Chem. Eng. 2017, 5, 10156.

[78] L. Zhang , P. Lu , Y. Luo , J. Y. Zheng , W. Ma , L.‐X. Ding , H. Wang , J. Mater. Chem. A 2021, 9, 9609.

[79] H. A. Altaleb , M. M. Abdul Hameed , B. M. Thamer , Mater. Today Chem. 2023, 30, 101564.

[80] M. Khan , A. B. Yousaf , M. Chen , C. Wei , X. Wu , N. Huang , Z. Qi , L. Li , J. Power Sources 2015, 282, 520.

[81] J. Sun , H. Ma , H. Jiang , L. Dang , Q. Lu , F. Gao , J. Mater. Chem. A 2015, 3, 15882.

[82] L. Dong , R. R. S. Gari , Z. Li , M. M. Craig , S. Hou , Carbon 2010, 48, 781.

[83] H. Yang , L. Geng , Y. Zhang , G. Chang , Z. Zhang , X. Liu , M. Lei , Y. He , Appl. Surf. Sci. 2019, 466, 385.

[84] G. V. Reddy , P. Raghavendra , B. Ankamwar , P. Sri Chandana , S. M. Senthil Kumar , L. S. Sarma , Mater. Chem. Front. 2017, 1, 757.

[85] Y. Lu , Y. Jiang , H. Wu , W. Chen , J. Phys. Chem. C 2013, 117, 2926.

[86] L. Lin , M. Yuan , Z. Sun , H. Li , C. Nan , G. Sun , S. Ma , Dalton Trans. 2018, 47, 15131. 30310897 10.1039/c8dt03175d

[87] G. Zhang , Z. Yang , W. Zhang , Y. Wang , J. Mater. Chem. A 2016, 4, 3316.

[88] J. Zhao , J. Shu , J. Wang , H. Yang , Z. Dong , S. Li , Nanoscale 2022, 14, 17392. 36382672 10.1039/d2nr04600h

[89] S. Li , S. Ma , Y. Zhang , L. Zhao , H. Yang , R. Jin , J. Colloid Interface Sci. 2021, 588, 384. 33422787 10.1016/j.jcis.2020.12.080

[90] X. Yuan , J. Li , C. Zhang , W. Yue , Electrochim. Acta 2020, 340, 135969.

[91] H. Yang , H. Zou , M. Chen , S. Li , J. Jin , J. Ma , Inorg. Chem. Front. 2017, 4, 1881.

[92] Y. Zhang , F. Gao , P. Song , J. Wang , J. Guo , Y. Shiraishi , Y. Du , ACS Sustainable Chem. Eng. 2019, 7, 3176.

[93] L. Yang , G. Li , R. Ma , S. Hou , J. Chang , M. Ruan , W. Cai , Z. Jin , W. Xu , G. Wang , J. Ge , C. Liu , W. Xing , Nano Res. 2021, 14, 2853.

[94] H. Huang , L. Ma , C. S. Tiwary , Q. Jiang , K. Yin , W. Zhou , P. M. Ajayan , Small 2017, 13, 1603013. 10.1002/smll.201603013 28026150

[95] X. Zhang , J. Zhu , C. S. Tiwary , Z. Ma , H. Huang , J. Zhang , Z. Lu , W. Huang , Y. Wu , ACS Appl. Mater. Interfaces 2016, 8, 10858. 27082661 10.1021/acsami.6b01580

[96] W. Huang , X. Y. Ma , H. Wang , R. Feng , J. Zhou , P. N. Duchesne , P. Zhang , F. Chen , N. Han , F. Zhao , J. Zhou , W. B. Cai , Y. Li , Adv. Mater. 2017, 29, 1703057. 10.1002/adma.201703057 28762572

[97] R. Yue , H. Wang , D. Bin , J. Xu , Y. Du , W. Lu , J. Guo , J. Mater. Chem. A 2015, 3, 1077.

[98] X. Fan , S. Zerebecki , R. Du , R. Hubner , G. Marzum , G. Jiang , Y. Hu , S. Barcikowki , S. Reichenberger , A. Eychmuller , Angew. Chem., Int. Ed. 2020, 59, 5706. 10.1002/anie.201913079 PMC7154742 31990450

[99] A. A. Ensafi , M. Jafari‐Asl , B. Rezaei , Electrochim. Acta 2014, 130, 397.

[100] R. Wang , L. Yang , X. Wang , Z. Sun , Y. Guo , M. Lou , H. Shi , P. Wen , X. Hu , Inorg. Chem. 2021, 60, 13736. 34436878 10.1021/acs.inorgchem.1c02111

[101] L. Li , J. Zhang , Y. Liu , W. Zhang , H. Yang , J. Chen , Q. Xu , ACS Sustainable Chem. Eng. 2013, 1, 527.

[102] R.‐X. Wang , J.‐J. Fan , Y.‐J. Fan , J.‐P. Zhong , L. Wang , S.‐G. Sun , X.‐C. Shen , Nanoscale 2014, 6, 14999. 25363456 10.1039/c4nr04140b

[103] J. Ma , L. Wang , X. Mu , Y. Cao , J. Colloid Interface Sci. 2015, 457, 102. 26164241 10.1016/j.jcis.2015.06.031

[104] T. H. Nguyen , D. Yang , B. Zhu , H. Lin , T. Ma , B. Jia , J. Mater. Chem. A 2021, 9, 7366.

[105] Y. Liu , J. Ding , F. Li , X. Su , Q. Zhang , G. Guan , F. Hu , J. Zhang , Q. Wang , Y. Jiang , B. Liu , H. B. Yang , Adv. Mater. 2023, 35, 2207114. 10.1002/adma.202207114 36205652

[106] N. Talukder , Y. Wang , B. B. Nunna , E. S. Lee , Carbon 2021, 185, 198.

[107] H. Xu , L. Ma , Z. Jin , J. Energy Chem. 2018, 27, 146.

[108] M. Kaur , M. Kaur , V. K. Sharma , Adv. Colloid Interface Sci. 2018, 259, 44. 30032930 10.1016/j.cis.2018.07.001

[109] J. Yang , R. Hubner , J. Zhang , H. Wan , Y. Zheng , H. Wang , H. Qi , L. He , Y. Li , A. A. Dubale , Y. Sun , Y. Liu , D. Peng , Y. Meng , Z. Zheng , J. Rossmeisl , W. Liu , Angew. Chem., Int. Ed. 2021, 60, 9590. 10.1002/anie.202015679 33554402

[110] H. Lv , L. Sun , D. Xu , B. Liu , Sci. Bull. 2020, 65, 1823. 10.1016/j.scib.2020.05.005 36659122

[111] J. Cheng , C. Lyu , G. Dong , Y. Liu , Y. Hu , B. Han , D. Geng , D. Zhao , Electrochim. Acta 2023, 454, 142364.

[112] X. Chen , J. Zhao , J. Lian , X. Wang , Green Chem. 2023, 25, 3198.

[113] R. Shi , J. Zhao , S. Liu , W. Sun , H. Li , P. Hao , Z. Li , J. Ren , Carbon 2018, 130, 185.

[114] H. Yang , S. Li , R. Jin , Z. Yu , G. Yang , J. Ma , Chem. Eng. J. 2020, 389, 124487.

[115] L. Sun , H. Lv , D. Xu , B. Liu , J. Mater. Chem. A 2020, 8, 15706.

[116] L. Zhao , X.‐L. Sui , J.‐Z. Li , J.‐J. Zhang , L.‐M. Zhang , G.‐S. Huang , Z.‐B. Wang , Appl. Catal. B 2018, 231, 224.

[117] L.‐S. Zhang , X.‐Q. Liang , W.‐G. Song , Z.‐Y. Wu , Phys. Chem. Chem. Phys. 2010, 12, 12055. 20725652 10.1039/c0cp00789g

[118] Y. Li , H. Li , Y. Zhao , D. Ji , P. Guo , G. Li , X. Zhao , Small 2023, 19, 2303065. 10.1002/smll.202303065 37480183

[119] J. Y. Damte , S.‐L. Lyu , E. G. Leggesse , J. C. Jiang , Phys. Chem. Chem. Phys. 2018, 20, 9355. 29564450 10.1039/C7CP07618E

[120] Y. Sun , C. Du , M. An , L. Du , Q. Tan , C. Liu , Y. Gao , G. Yin , J. Power Sources 2015, 300, 245.

[121] M. An , C. Du , L. Du , Y. Sun , Y. Wang , C. Chen , G. Han , G. Yin , Y. Gao , Chem. Phys. Lett. 2017, 687, 1.

[122] I. Y. Jeon , S. Zhang , L. Zhang , H. J. Choi , J. M. Seo , Z. Xia , L. Dai , J. B. Baek , Adv. Mater. 2013, 25, 6138. 24038522 10.1002/adma.201302753

[123] Z. Wang , P. Li , Y. Chen , J. He , W. Zhang , O. G. Schmidt , Y. Li , Nanoscale 2014, 6, 7281. 24850434 10.1039/c3nr05061k

[124] D. K. Perivoliotis , Y. Sato , K. Suenaga , N. Tagmatarchis , ACS Appl. Energy Mater. 2018, 1, 3869.

[125] K. Zhang , X. Chen , L. Wang , D. Zhang , Z. Xue , X. Zhou , X. Lu , Int. J. Hydrogen Energy 2018, 43, 15931.

[126] C. Shi , X. Maimaitiyiming , Int. J. Hydrogen Energy 2021, 46, 10247.

[127] Y. Jin , D. Han , W. Jia , G. Huang , F. Li , X. Chen , R. Li , M. Zheng , W. Gao , J. Electrochem. Soc. 2017, 164, F638.

[128] D. Chen , Z. He , S.‐e. Pei , L.‐a. Huang , H. Shao , Y. Jin , J. Wang , J. Alloys Compd. 2019, 785, 781.

[129] L. Meng , W. Liu , Y. Lu , Z. Liang , T. He , J. Li , H. Nan , S. Luo , J. Yu , J. Energy Chem. 2023, 81, 633.

[130] M. Li , Q. Jiang , M. Yan , Y. Wei , J. Zong , J. Zhang , Y. Wu , H. Huang , ACS Sustainable Chem. Eng. 2018, 6, 6644.

[131] J.‐P. Zhong , C. Hou , M.‐L. Sun , Z.‐Y. Yang , D.‐H. Chen , Y.‐J. Fan , W. Chen , H.‐G. Liao , S.‐G. Sun , J. Mater. Chem. A 2022, 10, 13876.

[132] Z. Wang , Z. Lin , S. Shen , W. Zhong , S. Cao , Chin. J. Catal. 2021, 42, 710.

[133] W. Huang , H. Wang , J. Zhou , J. Wang , P. N. Duchesne , D. Muir , P. Zhang , N. Han , F. Zhao , M. Zeng , J. Zhong , C. Jin , Y. Li , S. T. Lee , H. Dai , Nat. Commun. 2015, 6, 10035. 26602295 10.1038/ncomms10035 PMC4674678

[134] L. J. Moriau , M. Bele , A. Vižintin , F. Ruiz‐Zepeda , U. Petek , P. Jovanovič , M. Šala , M. Gaberšček , N. Hodnik , ACS Catal. 2019, 9, 11468.

[135] K. Zhang , H. Wang , J. Qiu , J. Wu , H. Wang , J. Shao , Y. Deng , L. Yan , Chem. Eng. J. 2021, 421, 127786.

[136] Y.‐W. Zhou , Y.‐F. Chen , K. Jiang , Z. Liu , Z.‐J. Mao , W.‐Y. Zhang , W.‐F. Lin , W.‐B. Cai , Appl. Catal. B 2021, 280, 119393.

[137] P. Xu , S. Zhao , T. Wang , W. Ji , Z. Chen , C.‐T. Au , J. Mater. Chem. A 2022, 10, 10150.

[138] K. Zhang , J. Qiu , J. Wu , Y. Deng , Y. Wu , L. Yan , J. Mater. Chem. A 2022, 10, 4254.

[139] T. Wu , Y. Ma , Z. Qu , J. Fan , Q. Li , P. Shi , Q. Xu , Y. Min , ACS Appl. Mater. Interfaces 2019, 11, 5136. 30648393 10.1021/acsami.8b20240

[140] J. Wang , X. You , C. Xiao , X. Zhang , S. Cai , W. Jiang , S. Guo , S. Cao , Z. Chen , Appl. Catal. B 2019, 259, 118060.

[141] Z. Gao , M. Li , J. Wang , J. Zhu , X. Zhao , H. Huang , J. Zhang , Y. Wu , Y. Fu , X. Wang , Carbon 2018, 139, 369.

[142] S. Li , H. Yang , H. Zou , M. Yang , X. Liu , J. Jin , J. Ma , J. Mater. Chem. A 2018, 6, 14717.

[143] Q. Zhang , Y. Li , H. He , H. Huang , ACS Sustainable Chem. Eng. 2022, 10, 8940.

[144] S. Ramakrishnan , M. Karuppannan , M. Vinothkannan , K. Ramachandran , O. J. Kwon , D. J. Yoo , ACS Appl. Mater. Interfaces 2019, 11, 12504. 30848889 10.1021/acsami.9b00192

[145] X. Du , W. Cai , Q. Zhang , L. Yang , H. He , ACS Appl. Nano Mater. 2021, 4, 9729.

[146] J. Duan , X. Zhang , W. Yuan , H. Chen , S. Jiang , X. Liu , Y. Zhang , L. Chang , Z. Sun , J. Du , J. Power Sources 2015, 285, 76.

[147] M. Yan , Q. Jiang , T. Zhang , J. Wang , L. Yang , Z. Lu , H. He , Y. Fu , X. Wang , H. Huang , J. Mater. Chem. A 2018, 6, 18165.

[148] Y. Wang , Y. Nian , A. N. Biswas , W. Li , Y. Han , J. G. Chen , Adv. Energy Mater. 2021, 11, 2002967.

[149] R. Fang , C. Lu , A. Chen , K. Wang , H. Huang , Y. Gan , C. Liang , J. Zhang , X. Tao , Y. Xia , W. Zhang , ChemSusChem 2020, 13, 1409. 31593617 10.1002/cssc.201902537

[150] M. Naguib , M. Kurtoglu , V. Presser , J. Lu , J. Niu , M. Heon , L. Hultman , Y. Gogotsi , M. W. Barsoum , Adv. Mater. 2011, 23, 4248. 21861270 10.1002/adma.201102306

[151] S. Xiao , Y. Zheng , X. Wu , M. Zhou , X. Rong , L. Wang , Y. Tang , X. Liu , L. Qiu , C. Cheng , Small 2022, 18, 2203281. 10.1002/smll.202203281 35989101

[152] A. Liu , X. Liang , X. Ren , W. Guan , M. Gao , Y. Yang , Q. Yang , L. Gao , Y. Li , T. Ma , Adv. Funct. Mater. 2020, 30, 2003437.

[153] J. Pang , R. G. Mendes , A. Bachmatiuk , L. Zhao , H. Q. Ta , T. Gemming , H. Liu , Z. Liu , M. H. Rummeli , Chem. Soc. Rev. 2019, 48, 72. 30387794 10.1039/c8cs00324f

[154] J. Zhang , Y. Zhao , X. Guo , C. Chen , C.‐L. Dong , R.‐S. Liu , C.‐P. Han , Y. Li , Y. Gogotsi , G. Wang , Nat. Catal. 2018, 1, 985.

[155] C. Yang , H. Huang , H. He , L. Yang , Q. Jiang , W. Li , Coord. Chem. Rev. 2021, 435, 213806.

[156] S. G. Peera , C. Liu , J. Shim , A. K. Sahu , T. G. Lee , M. Selvaraj , R. Koutavarapu , Ceram. Int. 2021, 47, 28106.

[157] M. Naguib , V. N. Mochalin , M. W. Barsoum , Y. Gogotsi , Adv. Mater. 2014, 26, 992. 24357390 10.1002/adma.201304138

[158] M. Alhabeb , K. Maleski , B. Anasori , P. Lelyukh , L. Clark , S. Sin , Y. Gogotsi , Chem. Mater. 2017, 29, 7633.

[159] J. L. Hart , K. Hantanasirisakul , A. C. Lang , B. Anasori , D. Pinto , Y. Pivak , J. T. van Omme , S. J. May , Y. Gogotsi , M. L. Taheri , Nat. Commun. 2019, 10, 522. 30705273 10.1038/s41467-018-08169-8 PMC6355901

[160] C. Y. Liu , E. Y. Li , ACS Appl. Mater. Interfaces 2019, 11, 1638. 30539632 10.1021/acsami.8b17600

[161] M.‐S. Cao , Y.‐Z. Cai , P. He , J.‐C. Shu , W.‐Q. Cao , J. Yuan , Chem. Eng. J. 2019, 359, 1265.

[162] Y. Zhao , J. Zhang , X. Guo , X. Cao , S. Wang , H. Liu , G. Wang , Chem. Soc. Rev. 2023, 52, 3215. 37073529 10.1039/d2cs00698g

[163] D. Zhao , Z. Chen , W. Yang , S. Liu , X. Zhang , Y. Yu , W. C. Cheong , L. Zheng , F. Ren , G. Ying , X. Cao , D. Wang , Q. Peng , G. Wang , C. Chen , J. Am. Chem. Soc. 2019, 141, 4086. 30699294 10.1021/jacs.8b13579

[164] X. Yang , Y. Zhang , Z. Fu , Z. Lu , X. Zhang , Y. Wang , Z. Yang , R. Wu , ACS Appl. Mater. Interfaces 2020, 12, 28206. 32463647 10.1021/acsami.0c06174

[165] Y. Wang , J. Wang , G. Han , C. Du , Q. Deng , Y. Gao , G. Yin , Y. Song , Ceram. Int. 2019, 45, 2411.

[166] J. Zhao , Z. Tu , S. H. Chan , J. Power Sources 2021, 488, 229434.

[167] C. Yang , T. Wang , C. Li , H. He , D. Liu , H. Huang , ACS Appl. Mater. Interfaces 2023, 15, 49195. 37843990 10.1021/acsami.3c10789

[168] H. Huang , D. Xiao , Z. Zhu , C. Zhang , L. Yang , H. He , J. You , Q. Jiang , X. Xu , Y. Yamauchi , Chem. Sci. 2023, 14, 9854. 37736638 10.1039/d3sc03735e PMC10510762

[169] Z. Lang , Z. Zhuang , S. Li , L. Xia , Y. Zhao , Y. Zhao , C. Han , L. Zhou , ACS Appl. Mater. Interfaces 2020, 12, 2400. 31868343 10.1021/acsami.9b17088

[170] J. Zhu , L. Xia , R. Yu , R. Lu , J. Li , R. He , Y. Wu , W. Zhang , X. Hong , W. Chen , Y. Zhao , L. Zhou , L. Mai , Z. Wang , J. Am. Chem. Soc. 2022, 144, 15529. 35943197 10.1021/jacs.2c03982

[171] C. Yang , Q. Jiang , H. Liu , L. Yang , H. He , H. Huang , W. Li , J. Mater. Chem. A 2021, 9, 15432.

[172] L. Durai , S. Badhulika , Fuel 2023, 352, 129058.

[173] V. M. Hong Ng , H. Huang , K. Zhou , P. S. Lee , W. Que , J. Z. Xu , L. B. Kong , J. Mater. Chem. A 2017, 5, 3039.

[174] X. Hui , X. Ge , R. Zhao , Z. Li , L. Yin , Adv. Funct. Mater. 2020, 30, 2005190.

[175] V. S. Navjyoti , V. Bhullar , V. Saxena , A. K. Debnath , A. Mahajan , Langmuir 2023, 39, 2995. 36786558 10.1021/acs.langmuir.2c02845

[176] J. Qin , H. Huang , Y. Xie , S. Pan , Y. Chen , L. Yang , Q. Jiang , H. He , Ceram. Int. 2022, 48, 15327.

[177] M. Chandran , A. Raveendran , A. Thomas , M. Vinoba , S. K. Jeong , M. Bhagiyalakshmi , Synth. Met. 2023, 293, 117260.

[178] P. Wang , H. Cui , C. Wang , Nano Energy 2019, 66, 104196.

[179] S. Xie , J. Shu , H. Huang , J. Li , R. Yue , J. Xu , J. Colloid Interface Sci. 2023, 639, 314. 36805756 10.1016/j.jcis.2023.02.081

[180] S. Chen , N. Liu , J. Zhong , R. Yang , B. Yan , L. Gan , P. Yu , X. Gui , H. Yang , D. Yu , Z. Zeng , G. Yang , Angew. Chem., Int. Ed. 2022, 61, 202209693. 10.1002/anie.202209693 36114595

[181] C. Yang , Q. Jiang , H. Huang , H. He , L. Yang , W. Li , ACS Appl. Mater. Interfaces 2020, 12, 23822. 32356656 10.1021/acsami.0c02806

[182] X. Zhang , J. Zhang , H. Cao , Y. Li , Appl. Catal. A 2019, 585, 117181.

[183] P. Zhang , C. Fan , R. Wang , C. Xu , J. Cheng , L. Wang , Y. Lu , P. Luo , Nanotechnology 2020, 31, 09LT01. 10.1088/1361-6528/ab5609 31711050

[184] Y. Z. Chen , M. Zhou , Y. F. Huang , Y.‐Y. Ma , L.‐Y. Yan , X.‐W. Zhou , X.‐Z. Ma , X.‐L. Zhao , C. Chen , J. Bai , D.‐H. Lin , Rare Met. 2022, 41, 3170.

[185] S. Li , L. Zhao , J. Shu , H. Niu , R. Li , J. Zhao , H. Yang , J. Jin , R. Jin , J. Colloid Interface Sci. 2022, 610, 944. 34863544 10.1016/j.jcis.2021.11.142

[186] W. Zhan , L. Ma , M. Gan , Mater. Today Chem. 2022, 26, 101041.

[187] J. Qin , H. Huang , J. Zhang , F. Zhu , L. Luo , C. Zhang , L. Yang , Q. Jiang , H. He , J. Mater. Chem. A 2023, 11, 2848.

[188] C. Yao , Q. Zhang , B. Cheng , Q. Chen , R. Wang , X. Zhou , Y. Ren , F. Wei , M. Zhang , J. Alloys Compd. 2023, 953, 169983.

[189] T. Y. Ma , J. L. Cao , M. Jaroniec , S. Z. Qiao , Angew. Chem., Int. Ed. 2016, 55, 1138. 10.1002/anie.201509758 26629779

[190] B. A. Catalysis A: GeneralChen , Z. Lu , S. Feng , Z. Zhou , C. Lu , ACS Energy Lett. 2023, 8, 1096.

[191] R. Wang , M. Li , K. Sun , Y. Zhang , J. Li , W. Bao , Small 2022, 18, 2201740. 10.1002/smll.202201740 35532321

[192] Y. Yoon , A. P. Tiwari , M. Choi , T. G. Novak , W. Song , H. Chang , T. Zyung , S. S. Lee , S. Jeon , K. S. An , Adv. Funct. Mater. 2019, 29, 1903443.

[193] X. Chen , X. Zhai , J. Hou , H. Cao , X. Yue , M. Li , L. Chen , Z. Liu , G. Ge , X. Guo , Chem. Eng. J. 2021, 420, 129832.

[194] W. Peng , J. Han , Y. R. Lu , M. Luo , T. S. Chan , M. Peng , Y. Tan , ACS Nano 2022, 16, 4116. 35187929 10.1021/acsnano.1c09841

[195] Z. Chen , B. Yu , J. Cao , X. Wen , M. Luo , S. Xing , D. Chen , C. Feng , G. Huang , Y. Jin , Electrochim. Acta 2021, 390, 138902.

[196] C. Ling , L. Shi , Y. Ouyang , Q. Chen , J. Wang , Adv. Sci. 2016, 3, 1600180. 10.1002/advs.201600180 PMC5102657 27980992

[197] Z. Wu , T. Shang , Y. Deng , Y. Tao , Q. H. Yang , Adv. Sci. 2020, 7, 1903077. 10.1002/advs.201903077 PMC7141041 32274307

[198] X. Zhuang , S. Zhang , Y. Tang , F. Yu , Z. Li , H. Pang , Coord. Chem. Rev. 2023, 490, 215208.

[199] C. Tang , B.‐Q. Li , Q. Zhang , L. Zhu , H.‐F. Wang , J.‐L. Shi , F. Wei , Adv. Funct. Mater. 2016, 26, 577.

[200] Q. Zhang , Y. Li , T. Chen , L. Li , S. Shi , C. Jin , B. Yang , S. Hou , J. Electroanal. Chem. 2021, 894, 115338.

[201] K. R. G. Lim , A. D. Handoko , L. R. Johnson , X. Meng , M. Lin , G. S. Subramanian , B. Anasori , Y. Gogotsi , A. Vojvodic , Z. W. Seh , ACS Nano 2020, 14, 16140. 33186028 10.1021/acsnano.0c08671

[202] D. S. Abraham , M. Chandran , M. Vinoba , R. Yamuna , M. Bhagiyalakshmi , Langmuir 2023, 39, 4756. 36943685 10.1021/acs.langmuir.3c00154

[203] M. Chandran , A. Raveendran , M. Vinoba , B. K. Vijayan , M. Bhagiyalakshmi , Ceram. Int. 2021, 47, 26847.

[204] M. Elancheziyan , M. Eswaran , C. E. Shuck , S. Senthilkumar , S. Elumalai , R. Dhanusuraman , V. K. Ponnusamy , Fuel 2021, 303, 121329.

[205] M. Sadhukhan , M. K. Kundu , T. Bhowmik , S. Barman , Int. J. Hydrogen Energy 2017, 42, 9371.

[206] H. Qian , S. Chen , Y. Fu , X. Wang , J. Power Sources 2015, 300, 41.

[207] H. Qian , H. Huang , X. Wang , J. Power Sources 2015, 275, 734.

[208] W. Zhang , Q. Yao , G. Jiang , C. Li , Y. Fu , X. Wang , A. Yu , Z. Chen , ACS Catal. 2019, 9, 11603.

[209] F. Zhang , Z. Wang , K. Q. Xu , J. Xia , Q. Liu , Z. Wang , Int. J. Hydrogen Energy 2018, 43, 16302.

[210] H. Wang , Y. Chen , W. Xie , X. Han , Q. Feng , R. Jiang , H. Shang , F. Zhang , L. Gao , Z. Wang , Int. J. Electrochem. Sci. 2019, 14, 7961.

[211] F. Xie , L. Ma , M. Gan , H. He , L. Hu , M. Jiang , H. Zhang , J. Power Sources 2019, 420, 73.

[212] M. Chen , Y. Duan , F. chen , W. Zheng , W. Yang , M. Tian , Energy Fuels 2023, 37, 2350.

[213] Q. Zhang , J. Weng , J. Xu , J. Phys. Chem. C 2021, 125, 18717.

[214] T. Wu , J. Fan , Q. Li , P. Shi , Q. Xu , Y. Min , Adv. Energy Mater. 2017, 8, 1701799.

[215] L. Hui , X. Zhang , Y. Xue , X. Chen , Y. Fang , C. Xing , Y. Liu , X. Zheng , Y. Du , C. Zhang , F. He , Y. Li , J. Am. Chem. Soc. 2022, 144, 1921. 35044172 10.1021/jacs.1c12310

[216] L. Hui , Y. Xue , C. Xing , Y. Liu , Y. Du , Y. Fang , H. Yu , B. Huang , Y. Li , Adv. Sci. 2022, 9, 2104991. 10.1002/advs.202104991 PMC9165484 35393786

[217] X. Zhang , L. Hui , D. Yan , J. Li , X. Chen , H. Wu , Y. Li , Angew. Chem., Int. Ed. 2023, 62, 202308968. 10.1002/anie.202308968 37581223

[218] Y. Zheng , J. Liu , J. Liang , M. Jaroniec , S. Z. Qiao , Energy Environ. Sci. 2012, 5, 6717.

[219] P. Suja , J. John , T. P. D. Rajan , G. M. Anilkumar , T. Yamaguchi , S. C. Pillai , U. S. Hareesh , J. Mater. Chem. A 2023, 11, 8599.

[220] W. Niu , Y. Yang , ACS Energy Lett. 2018, 3, 2796.

[221] E. Kroke , M. Schwarz , E. Horath‐Bordon , P. Kroll , B. Noll , A. D. Norman , New J. Chem. 2002, 26, 508.

[222] M. Inagaki , T. Tsumura , T. Kinumoto , M. Toyoda , Carbon 2019, 141, 580.

[223] J. Fu , K. Liu , K. Jiang , H. Li , P. An , W. Li , N. Zhang , H. Li , X. Xu , H. Zhou , D. Tang , X. Wang , X. Qiu , M. Liu , Adv. Sci. 2019, 6, 1900796. 10.1002/advs.201900796 PMC6755511 31559128

[224] C. Lu , X. Chen , ACS Nano 2021, 15, 18777. 34723464 10.1021/acsnano.1c06454

[225] W. Chen , Z.‐C. He , G.‐B. Huang , C.‐L. Wu , W.‐F. Chen , X.‐H. Liu , Chem. Eng. J. 2019, 359, 244.

[226] M.‐M. Fang , J.‐X. Shao , X.‐G. Huang , J.‐Y. Wang , W. Chen , J. Mater. Sci. Technol. 2020, 56, 133.

[227] Y. Yu , H. Huang , Chem. Eng. J. 2023, 453, 139755.

[228] Y. Wang , L. Liu , T. Ma , Y. Zhang , H. Huang , Adv. Funct. Mater. 2021, 31, 2102540.

[229] X. Zou , Z. Sun , Y. H. Hu , J. Mater. Chem. A 2020, 8, 21474.

[230] J. Zhang , J. Lang , Y. Wei , Q. Zheng , L. Liu , Y.‐H. Hu , B. Zhou , C. Yuan , M. Long , Appl. Catal. B 2021, 298, 120522.

[231] M. Zhu , C. Zhai , M. Sun , Y. Hu , B. Yan , Y. Du , Appl. Catal. B 2017, 203, 108.

[232] P. Chandrasekharan Meenu , S. P. Datta , S. A. Singh , S. Dinda , C. Chakraborty , S. Roy , J. Power Sources 2020, 461, 228150.

[233] Q. Zhang , Z. Yang , J. Yang , X. Yu , Y. Ling , Y. Zhang , W. Cai , H. Cheng , Chem. Commun. 2018, 54, 9282. 10.1039/c8cc03752c 29896585

[234] H. Sun , H. Huang , C. Hu , Y. Yan , Y. Hu , S.‐J. Liu , H.‐R. Wen , Int. J. Hydrogen Energy 2022, 47, 22796.

[235] S. Bama Sundararaj , S. Tamilarasan , K. Kadirvelu , S. Thangavelu , Appl. Surf. Sci. 2023, 612, 155785.

[236] S. Kumar , Y.‐P. Fu , Electrochim. Acta 2023, 447, 142161.

[237] P. C. Nagajyothi , K. Yoo , I. Y. Eom , J. Shim , Ceram. Int. 2022, 48, 11623.

[238] I. S. Pieta , A. Rathi , P. Pieta , R. Nowakowski , M. Hołdynski , M. Pisarek , A. Kaminska , M. B. Gawande , R. Zboril , Appl. Catal. B 2019, 244, 272.

[239] Z. Chen , W. Chen , G. Liao , X. Li , J. Wang , Y. Tang , L. Li , J. Hazard. Mater. 2022, 428, 128222. 35032960 10.1016/j.jhazmat.2022.128222

[240] M. Li , Q. Ye , S. Hou , J. Yang , B. Chi , Y. Deng , X. Tian , J. Mater. Chem. A 2023, 11, 8730.

[241] L. Du , B. Gao , S. Xu , Q. Xu , Nat. Commun. 2023, 14, 2278. 37080974 10.1038/s41467-023-38012-8 PMC10119309

[242] J. Xu , Y. Chen , M. Chen , J. Wang , L. Wang , Chem. Eng. J. 2022, 442, 136208.

[243] I. Khan , S. Khan , S. Y. Wu , H. T. Chen , A. Zada , L. Linlin , A. Ismail , S. Ali , F. Raziq , M. Haider , J. Khan , S. Ullah , S. P. Ju , S. Wang , Small 2023, 19, 2208179. 10.1002/smll.202208179 36935369

[244] S.‐F. Ng , X. Chen , J. J. Foo , M. Xiong , W.‐J. Ong , Chin. J. Catal. 2023, 47, 150.

[245] X. Wang , M. Sun , Y. Guo , J. Hu , M. Zhu , J. Colloid Interface Sci. 2020, 558, 38. 31586742 10.1016/j.jcis.2019.09.085

[246] D. Fang , L. Yang , G. Yang , G. Yi , Y. Feng , P. Shao , H. Shi , K. Yu , D. You , X. Luo , Int. J. Hydrogen Energy 2020, 45, 21483.

[247] W. Zhang , Y. Fu , J. Wang , X. Wang , Adv. Mater. Interfaces 2017, 4, 1601219.

[248] Z. Li , R. Lin , Z. Liu , D. Li , H. Wang , Q. Li , Electrochim. Acta 2016, 191, 606.

[249] C.‐Z. Li , Z.‐B. Wang , X.‐L. Sui , L.‐M. Zhang , D.‐M. Gu , Carbon 2015, 93, 105.

[250] W. Zhang , H. Huang , F. Li , K. Deng , X. Wang , J. Mater. Chem. A 2014, 2, 19084.

[251] X. Liang , F. Dong , Z. Tang , Q. Wang , Int. J. Hydrogen Energy 2021, 46, 39645.

[252] M. Eswaran , R. Dhanusuraman , P.‐C. Tsai , V. K. Ponnusamy , Fuel 2019, 251, 91.

[253] R. H. Baughman , H. Eckhardt , M. Kertesz , J. Chem. Phys. 1987, 87, 6687.

[254] G. Li , Y. Li , H. Liu , Y. Guo , Y. Li , D. Zhu , Chem. Commun. 2010, 46, 3256. 10.1039/b922733d 20442882

[255] X. Fu , X. Zhao , T. B. Lu , M. Yuan , M. Wang , Angew. Chem., Int. Ed. 2023, 62, 202219242. 10.1002/anie.202219242 36723492

[256] F. He , Y. Li , CCS Chem. 2023, 5, 72.

[257] H. Li , J. H. Lim , Y. Lv , N. Li , B. Kang , J. Y. Lee , Chem. Rev. 2023, 123, 4795. 36921251 10.1021/acs.chemrev.2c00729

[258] J. Li , X. Gao , L. Zhu , M. N. Ghazzal , J. Zhang , C.‐H. Tung , L.‐Z. Wu , Energy Environ. Sci. 2020, 13, 1326.

[259] N. Wang , J. He , K. Wang , Y. Zhao , T. Jiu , C. Huang , Y. Li , Adv. Mater. 2019, 31, 1803202. 10.1002/adma.201803202 31448452

[260] R. Liu , X. Gao , J. Zhou , H. Xu , Z. Li , S. Zhang , Z. Xie , J. Zhang , Z. Liu , Adv. Mater. 2017, 29, 1604665. 10.1002/adma.201604665 28251693

[261] R. Matsuoka , R. Sakamoto , K. Hoshiko , S. Sasaki , H. Masunaga , K. Nagashio , H. Nishihara , J. Am. Chem. Soc. 2017, 139, 3145. 28199105 10.1021/jacs.6b12776

[262] X. Gao , Y. Zhu , D. Yi , J. Zhou , S. Zhang , C. Yin , F. Ding , S. Zhang , X. Yi , J. Wang , L. Tong , Y. Han , Z. Liu , J. Zhang , Sci. Adv. 2018, 4, eaat6378. 29984309 10.1126/sciadv.aat6378 PMC6035041

[263] B. Song , M. Chen , G. Zeng , J. Gong , M. Shen , W. Xiong , C. Zhou , X. Tang , Y. Yang , W. Wang , J. Hazard. Mater. 2020, 398, 122957. 32474321 10.1016/j.jhazmat.2020.122957

[264] Y. Gao , Y. Xue , T. Liu , Y. Liu , C. Zhang , C. Xing , F. He , Y. Li , Adv. Sci. 2021, 8, 2102777. 10.1002/advs.202102777 PMC8564434 34494718

[265] G. Tian , Z. Qi , W. Ma , Y. Wang , ChemistrySelect 2017, 2, 2311. 28966970 10.1002/slct.201601874 PMC5613985

[266] C. Zhang , Y. Zhang , H. Xiao , J. Zhang , L. Li , L. Wang , Q. Bai , M. Liu , Z. Wang , N. Sui , Colloid Surf., A 2021, 612, 125960.

[267] L. Hui , Y. Xue , C. Xing , Y. Liu , Y. Du , Y. Fang , H. Yu , C. Zhang , F. He , Y. Li , Nano Energy 2022, 95, 106984.

[268] B. Liu , L. Xu , Y. Zhao , J. Du , N. Yang , D. Wang , J. Mater. Chem. A 2021, 9, 19298.

[269] C. Xing , Y. Xue , B. Huang , H. Yu , L. Hui , Y. Fang , Y. Liu , Y. Zhao , Z. Li , Y. Li , Angew. Chem., Int. Ed. 2019, 58, 13897. 10.1002/anie.201905729 31309671

[270] N. Wang , X. Li , Z. Tu , F. Zhao , J. He , Z. Guan , C. Huang , Y. Yi , Y. Li , Angew. Chem., Int. Ed. 2018, 57, 3968. 10.1002/anie.201800453 29397008

[271] N. Wang , J. He , Z. Tu , Z. Yang , F. Zhao , X. Li , C. Huang , K. Wang , T. Jiu , Y. Yi , Y. Li , Angew. Chem., Int. Ed. 2017, 56, 10740. 10.1002/anie.201704779 28691245

[272] G. Fan , F. Li , D. G. Evans , X. Duan , Chem. Soc. Rev. 2014, 43, 7040. 25001024 10.1039/c4cs00160e

[273] H. Boumeriame , E. S. Da Silva , A. S. Cherevan , T. Chafik , J. L. Faria , D. Eder , J. Energy Chem. 2022, 64, 406.

[274] Y. Wang , D. Yan , S. El Hankari , Y. Zou , S. Wang , Adv. Sci. 2018, 5, 1800064. 10.1002/advs.201800064 PMC6096997 30128233

[275] Y. Wang , M. Zhang , Y. Liu , Z. Zheng , B. Liu , M. Chen , G. Guan , K. Yan , Adv. Sci. 2023, 10, 2207519. 10.1002/advs.202207519 PMC10161082 36866927

[276] H. Yi , S. Liu , C. Lai , G. Zeng , M. Li , X. Liu , B. Li , X. Huo , L. Qin , L. Li , M. Zhang , Y. Fu , Z. An , L. Chen , Adv. Energy Mater. 2021, 11, 2002863.

[277] A. Hameed , M. Batool , Z. Liu , M. A. Nadeem , R. Jin , ACS Energy Lett. 2022, 7, 3311.

[278] B. Liu , X. Wang , S. Wang , H.‐Q. Peng , T. Xiao , G. Liu , S. Bai , Y. Zhao , W. Zhang , Y.‐F. Song , Mater. Today Energy 2022, 28, 101082.

[279] H. Chi , J. Lin , S. Kuang , M. Li , H. Liu , Q. Fan , T. Yan , S. Zhang , X. Ma , J. Energy Chem. 2023, 85, 267.

[280] J. Shi , H. He , Y. Guo , F. Ji , J. Li , Y. Zhang , C. Deng , L. Fan , W. Cai , J. Energy Chem. 2023, 85, 76.

[281] M. Li , X. Deng , Y. Liang , K. Xiang , D. Wu , B. Zhao , H. Yang , J.‐L. Luo , X.‐Z. Fu , J. Energy Chem. 2020, 50, 314.

[282] Y. Zhang , X. Wu , G. Fu , F. Si , X.‐Z. Fu , J.‐L. Luo , Int. J. Hydrogen Energy 2022, 47, 17150.

[283] Y. Wang , D. Zhang , M. Tang , S. Xu , M. Li , Electrochim. Acta 2010, 55, 4045.

[284] B. Ballarin , A. Mignani , E. Scavetta , M. Giorgetti , D. Tonelli , E. Boanini , C. Mousty , V. Prevot , Langmuir 2012, 28, 15065. 23025480 10.1021/la302938t

[285] W. Song , Y. Xu , X. Xie , C. Li , W. Zhu , Q. Xiang , W. Chen , N. Tang , L. Wang , ACS Appl. Mater. Interfaces 2023, 15, 27253. 37216444 10.1021/acsami.3c01541

[286] Y. Han , J. Wu , Q. Li , X. Yang , T. Li , Q. Wang , X. Wu , Colloid Surf., A 2023, 669, 131497.

[287] L. Li , Y. Yang , Y. Wang , M. Liang , Y. Huang , J. Mater. Res. Technol. 2020, 9, 5463.

[288] H. Y. Zhu , C. D. Gu , X. Ge , J. P. Tu , Electrochim. Acta 2016, 222, 938.

[289] F. Fathirad , E. Sadeghi , Fuel 2024, 358, 130130.

[290] Z. Wang , F. Zhang , H. Zou , Y. Yuan , H. Wang , J. Xia , Z. Wang , J. Electroanal. Chem. 2018, 818, 198.

[291] C. Xing , J. Zhang , J. Jing , J. Li , F. Shi , Chem. Eng. J. 2019, 370, 120.

[292] L. Li , Y. Yu , G. J. Ye , Q. Ge , X. Ou , H. Wu , D. Feng , X. H. Chen , Y. Zhang , Nat. Nanotechnol. 2014, 9, 372. 24584274 10.1038/nnano.2014.35

[293] H. Liu , A. T. Neal , Z. Zhu , Z. Luo , X. Xu , D. Tomanek , P. D. Ye , ACS Nano 2014, 8, 4033. 24655084 10.1021/nn501226z

[294] F. Shi , Z. Geng , K. Huang , Q. Liang , Y. Zhang , Y. Sun , J. Cao , S. Feng , Adv. Sci. 2018, 5, 1800575. 10.1002/advs.201800575 PMC6096989 30128261

[295] T. Yin , L. Long , X. Tang , M. Qiu , W. Liang , R. Cao , Q. Zhang , D. Wang , H. Zhang , Adv. Sci. 2020, 7, 2001431. 10.1002/advs.202001431 PMC7539224 33042754

[296] H. Liu , K. Hu , D. Yan , R. Chen , Y. Zou , H. Liu , S. Wang , Adv. Mater. 2018, 30, 1800295. 10.1002/adma.201800295 29782658

[297] Z. He , R. Liu , C. Xu , Y. Lai , W. Shan , J. Liu , Appl. Catal. B 2021, 285, 119775.

[298] M. Vanni , M. Bellini , S. Borsacchi , L. Calucci , M. Caporali , S. Caporali , F. d'Acapito , M. Geppi , A. Giaccherini , A. Ienco , G. Manca , A. M. Mio , G. Nicotra , W. Oberhauser , M. Serrano‐Ruiz , M. Banchelli , F. Vizza , M. Peruzzini , J. Am. Chem. Soc. 2021, 143, 10088. 34185506 10.1021/jacs.1c01754 PMC9295127

[299] M. Vanni , M. Serrano‐Ruiz , F. Telesio , S. Heun , M. Banchelli , P. Matteini , A. M. Mio , G. Nicotra , C. Spinella , S. Caporali , A. Giaccherini , F. D'Acapito , M. Caporali , M. Peruzzini , Chem. Mater. 2019, 31, 5075. 31656368 10.1021/acs.chemmater.9b00851 PMC6804426

[300] X. Wang , L. Bai , J. Lu , X. Zhang , D. Liu , H. Yang , J. Wang , P. K. Chu , S. Ramakrishna , X. F. Yu , Angew. Chem., Int. Ed. 2019, 58, 19060. 10.1002/anie.201911696 31589358

[301] S. Seo , H. U. Lee , S. C. Lee , Y. Kim , H. Kim , J. Bang , J. Won , Y. Kim , B. Park , J. Lee , Sci. Rep. 2016, 6, 23736. 27026070 10.1038/srep23736 PMC4812320

[302] C. D. Zhang , J. C. Lian , W. Yi , Y. H. Jiang , L. W. Liu , H. Hu , W. D. Xiao , S. X. Du , L. L. Sun , H. J. Gao , J. Phys. Chem. C 2009, 113, 18823.

[303] Q. Zhou , Q. Chen , Y. Tong , J. Wang , Angew. Chem., Int. Ed. 2016, 55, 11437. 10.1002/anie.201605168 27529543

[304] D.‐H. Kang , M. H. Jeon , S. K. Jang , W.‐Y. Choi , K. N. Kim , J. Kim , S. Lee , G. Y. Yeom , J.‐H. Park , ACS Photonics 2017, 4, 1822.

[305] W. Cai , T. Cai , L. He , F. Chu , X. Mu , L. Han , Y. Hu , B. Wang , W. Hu , J. Hazard. Mater. 2020, 387, 121971. 31918053 10.1016/j.jhazmat.2019.121971

[306] W. Cai , Y. Hu , Y. Pan , X. Zhou , F. Chu , L. Han , X. Mu , Z. Zhuang , X. Wang , W. Xing , J. Colloid Interface Sci. 2020, 561, 32. 31812865 10.1016/j.jcis.2019.11.114

[307] S. Li , Y. Zhang , H. Huang , J. Energy Chem. 2022, 67, 745.

[308] K. Kim , H. Ahn , M. J. Park , ACS Appl. Mater. Interfaces 2017, 9, 30278. 28853541 10.1021/acsami.7b10821

[309] Y. Kwon , Y. Kim , J. W. Hong , Y. Whang , S. Kim , D. H. Wi , H. R. Byon , S. W. Han , J. Mater. Chem. A 2020, 8, 25842.

[310] M. Sarno , E. Ponticorvo , D. Scarpa , Chem. Eng. J. 2019, 377, 120600.

[311] S. Su , C. Zhang , L. Yuwen , X. Liu , L. Wang , C. Fan , L. Wang , Nanoscale 2016, 8, 602. 26645896 10.1039/c5nr06077j

[312] W. Qiao , L. Yu , J. Chang , F. Yang , L. Feng , Chin. J. Catal. 2023, 51, 113.

[313] Y. Zhao , X. Zheng , P. Gao , H. Li , Mater. Horiz. 2023, 10, 3948. 37466487 10.1039/d3mh00462g

[314] L. Yuwen , F. Xu , B. Xue , Z. Luo , Q. Zhang , B. Bao , S. Su , L. Weng , W. Huang , L. Wang , Nanoscale 2014, 6, 5762. 24658079 10.1039/c3nr06084e

[315] H. Zhang , J. He , C. Zhai , M. Zhu , Chin. Chem. Lett. 2019, 30, 2338.

[316] S. Y. Ma , H. H. Li , B. C. Hu , X. Cheng , Q. Q. Fu , S. H. Yu , J. Am. Chem. Soc. 2017, 139, 5890. 28362492 10.1021/jacs.7b01482

[317] G. You , J. Jiang , M. Li , L. Li , D. Tang , J. Zhang , X. C. Zeng , R. He , ACS Catal. 2017, 8, 132.

[318] J. Wang , Z. Pei , J. Liu , M. Hu , Y. Feng , P. Wang , H. Wang , N. Nie , Y. Wang , C. Zhi , Y. Huang , Nano Energy 2019, 65, 104052.

[319] S. Li , J. Wang , X. Lin , G. Xie , Y. Huang , X. Liu , H. J. Qiu , Adv. Funct. Mater. 2020, 31, 2007129.

[320] C. Y. Ahn , J. E. Park , S. Kim , O. H. Kim , W. Hwang , M. Her , S. Y. Kang , S. Park , O. J. Kwon , H. S. Park , Y. H. Cho , Y. E. Sung , Chem. Rev. 2021, 121, 15075. 34677946 10.1021/acs.chemrev.0c01337

[321] T. Lu , H. Wang , Nano Res. 2022, 15, 9764. 10.1007/s12274-021-3949-z PMC8727479 35003529

[322] M. Zhang , C. Lai , B. Li , S. Liu , D. Huang , F. Xu , X. Liu , L. Qin , Y. Fu , L. Li , H. Yi , L. Chen , Small 2021, 17, 2007113. 10.1002/smll.202007113 34047018

[323] J. Xia , B. Wang , J. Di , Y. Li , S.‐Z. Yang , H. Li , S. Guo , Mater. Today 2022, 53, 217.

[324] P. Zhu , X. Xiong , D. Wang , Nano Res. 2022, 15, 5792.

[325] R. Li , D. Wang , Nano Res. 2022, 15, 6888.

[326] Y. T. Kim , K. Ohshima , K. Higashimine , T. Uruga , M. Takata , H. Suematsu , T. Mitani , Angew. Chem., Int. Ed. 2006, 45, 407. 10.1002/anie.200501792 16342218

[327] A. Cuesta , J. Am. Chem. Soc. 2006, 128, 13332. 17031926 10.1021/ja0644172

[328] Z. Zhang , J. Liu , J. Wang , Q. Wang , Y. Wang , K. Wang , Z. Wang , M. Gu , Z. Tang , J. Lim , T. Zhao , F. Ciucci , Nat. Commun. 2021, 12, 5235. 34475400 10.1038/s41467-021-25562-y PMC8413426

[329] A. R. Poerwoprajitno , L. Gloag , J. Watt , S. Cheong , X. Tan , H. Lei , H. A. Tahini , A. Henson , B. Subhash , N. M. Bedford , B. K. Miller , P. B. O'Mara , T. M. Benedetti , D. L. Huber , W. Zhang , S. C. Smith , J. J. Gooding , W. Schuhmann , R. D. Tilley , Nat. Catal. 2022, 5, 231.

